# Mercury Isotope Fractionation during Dark Abiotic Reduction of Hg(II) by Dissolved, Surface-Bound, and Structural Fe(II)

**DOI:** 10.1021/acs.est.3c03703

**Published:** 2023-09-25

**Authors:** Lorenz Schwab, Niklas Gallati, Sofie M. Reiter, Richard L. Kimber, Naresh Kumar, David S. McLagan, Harald Biester, Stephan M. Kraemer, Jan G. Wiederhold

**Affiliations:** † Department of Environmental Geosciences, Centre for Microbiology and Environmental Systems Science, University of Vienna, Josef-Holaubek-Platz 2, 1090 Vienna, Austria; ‡ Doctoral School in Microbiology and Environmental Science, University of Vienna, 1030 Vienna, Austria; § Environmental Engineering Institute IIE-ENAC, Soil Biogeochemistry Laboratory, École Polytechnique Fédérale de Lausanne (EPFL), Route des Ronquos 86, 1951 Sion, Switzerland; ∥ Soil Chemistry and Chemical Soil Quality Group, Department of Environmental Sciences, University of Wageningen, Droevendaalsesteeg 3a, 6708 Wageningen, Netherlands; ⊥ Environmental Geochemistry Group, Institute of Geoecology, Technische Universität Braunschweig, Langer Kamp 19c, 38106 Braunschweig, Germany; # Department of Geological Sciences and Geological Engineering, Queen’s University, Kingston, Ontario K7L 3N6, Canada; ∇ School of Environmental Studies, Queen’s University, Kingston, Ontario K7L 3N6, Canada

**Keywords:** mercury, isotopes, redox processes, reduction, process tracing, Rayleigh fractionation

## Abstract

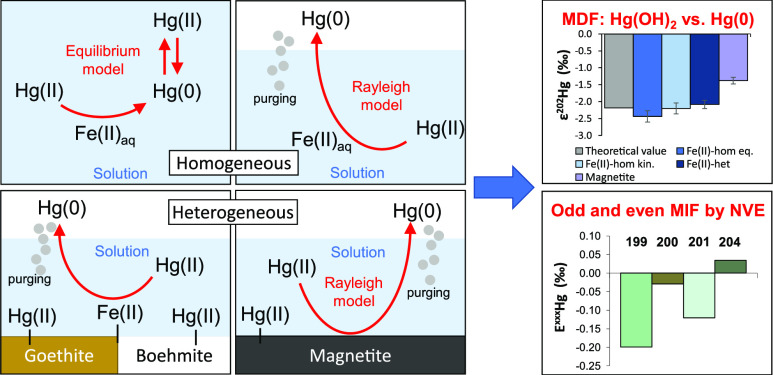

Stable mercury (Hg) isotope ratios are an emerging tracer for biogeochemical transformations in environmental systems, but their application requires knowledge of isotopic enrichment factors for individual processes. We investigated Hg isotope fractionation during dark, abiotic reduction of Hg(II) by dissolved iron(Fe)(II), magnetite, and Fe(II) sorbed to boehmite or goethite by analyzing both the reactants and products of laboratory experiments. For homogeneous reduction of Hg(II) by dissolved Fe(II) in continuously purged reactors, the results followed a Rayleigh distillation model with enrichment factors of −2.20 ± 0.16‰ (ε^202^Hg) and 0.21 ± 0.02‰ (E^199^Hg). In closed system experiments, allowing reequilibration, the initial kinetic fractionation was overprinted by isotope exchange and followed a linear equilibrium model with −2.44 ± 0.17‰ (ε^202^Hg) and 0.34 ± 0.02‰ (E^199^Hg). Heterogeneous Hg(II) reduction by magnetite caused a smaller isotopic fractionation (−1.38 ± 0.07 and 0.13 ± 0.01‰), whereas the extent of isotopic fractionation of the sorbed Fe(II) experiments was similar to the kinetic homogeneous case. Small mass-independent fractionation of even-mass Hg isotopes with 0.02 ± 0.003‰ (E^200^Hg) and ≈ −0.02 ± 0.01‰ (E^204^Hg) was consistent with theoretical predictions for the nuclear volume effect. This study contributes significantly to the database of Hg isotope enrichment factors for specific processes. Our findings show that Hg(II) reduction by dissolved Fe(II) in open systems results in a kinetic MDF with a larger ε compared to other abiotic reduction pathways, and combining MDF with the observed MIF allows the distinction from photochemical or microbial Hg(II) reduction pathways.

## Abstract 2

Redox processes strongly affect the environmental fate of mercury (Hg). We report mass-dependent and mass-independent Hg isotope fractionation during Hg(II) reduction by Fe(II) to better constrain isotopes as a process tracer.

## 1 Introduction

The toxic pollutant mercury (Hg) can occur in the environment in the oxidation states Hg(II), Hg(I), and Hg(0), and its chemical speciation governs the behavior and fate during biogeochemical cycling.^1,2^ In particular, redox transformations between oxidized Hg(II) and elemental Hg(0) are crucial in the global Hg cycle and largely determine Hg emissions from terrestrial and aquatic ecosystems to the atmosphere,^3,4^ where it can be transported over long distances and remain in circulation sufficiently long for global transport.^5−7^

Reduction of Hg(II) to Hg(0) in photic environments is mainly controlled by photoreduction,^8,9^ but in the absence of light, the reduction can proceed through biotic or abiotic pathways. In subsurface environments, such as groundwater, sediments, and hydromorphic soils, the interaction of Hg(II) with mineral surfaces plays a key role in determining its mobility and bioavailability. In situ formation of Hg(0) has been reported previously in contaminated groundwater^10−12^ and hydromorphic soils.^13^ Divalent mercury was shown to be reduced by dissolved Fe(II),^14,15^ surface-bound Fe(II) species,^14,16^ Fe(II)-bearing clays,^17^ and several Fe(II)-bearing minerals like siderite (FeCO_3_),^15,18^ mackinawite (FeS),^19,20^ and vivianite (Fe_3_(PO_4_)_2_),^21^ magnetite,^22−25^ and green rust.^25−27^ While aluminum (Al) and γ-alumina have an inhibitory effect on metal reduction rates,^14,28^ an enhancement of electron transfer from Fe(II) in the presence of iron (oxyhydr)oxide minerals has been reported in several systems.^28−30^

Mercury isotope ratios in natural samples show large variations caused by mass-dependent (MDF) and mass-independent (MIF) fractionation, which makes stable Hg isotopes a powerful multidimensional tool for tracing processes in Hg biogeochemical cycles.^31,32^ The application of Hg stable isotopes as a process tracer in complex biogeochemical environments relies on the determination of fractionation factors and characteristic MDF and MIF for individual transformation processes. Several studies examined isotope fractionation for different Hg(II) reduction pathways including microbial reduction,^33−35^ photoreduction in the presence of organic ligands,^36−40^ or dark abiotic reduction.^41^ In all of these experiments, the produced Hg(0) was continuously removed from the reactor and found to be enriched in light isotopes with the data following a Rayleigh-type fractionation. Recently, fractionation of Hg isotopes after partial Hg(II) reduction by siderite and green rust in closed system experiments was reported to follow an equilibrium fractionation model because of the rapid attainment of isotopic equilibrium by isotope exchange between Hg(II) and Hg(0) in solution.^18^ Similarly, the enrichment of heavy isotopes in Hg(II) during dark abiotic oxidation of Hg(0) to Hg(II) was explained by isotopic equilibrium by isotope exchange between Hg(II) and Hg(0).^42^

While the kinetics of Hg(II) reduction by ferrous iron have been described for systems with all reactants and products in the dissolved phase (homogeneous) and in the presence of a solid phase (heterogeneous), information on Hg isotope fractionation during these reactions, and especially the relative influence of kinetic and equilibrium fractionation mechanisms, is still lacking.

In this study, we investigated Hg stable isotope fractionation during the dark abiotic reduction of Hg(II) by dissolved Fe(II), Fe(II) bound to goethite and boehmite, and structural Fe(II) in magnetite by measuring the isotopic composition of reactant Hg(II) remaining in reactors and produced Hg(0) captured in oxidizing traps. Data on Hg isotope fractionation factors and mechanisms during both homogeneous and heterogeneous Hg(II) reduction by Fe(II) is provided. The influence of the presence of chloride (Cl^–^) and mineral surfaces on the isotope fractionation by Fe(II) was investigated in kinetically controlled reduction experiments in which the produced Hg(0) was immediately removed by continuous purging of the reactor. Additionally, the potential overprinting of kinetic isotope effects by equilibrium isotope fractionation was investigated by using a closed system approach allowing for isotope exchange between Hg(II) and the produced Hg(0) in solution before transferring Hg(0) to oxidizing traps.

## 2 Methods

### 2.1 Reagents and Mineral Synthesis

The Fe(II)Cl_2_-stock solution used in the experiments and for mineral synthesis was purified by precipitating Fe(III)-impurities following a published protocol^43^ (Section S1). Total Fe and Fe(II) concentrations were determined by ferrozine assay^44,45^ using a multiplate reader (Tecan Infinite 200 Pro). Boehmite (γ-alumina) was purchased from Sasol Chemicals and used without further treatment. Goethite was synthesized from an alkaline Fe(III)-system.^46^ Magnetite was synthesized biogenically by transforming 2-line-ferrihydrite using *Geobacter sulfurreducens* adjusting a published procedure.^47^ Detailed protocols for mineral synthesis are specified in the Supporting Information (SI, Section S2). The specific surface area of the minerals was obtained from Brunauer–Emmett–Teller (BET) analysis (Quantachrome 95 Nova 2000e). Powder X-ray diffraction (XRD) was used for the characterization of the freshly synthesized minerals (Rigaku Miniflex 600).

### 2.2 Species Calculation of Initial Solutions

Speciation distribution in experimental solutions was calculated using Visual MINTEQ 3.1^48^ using the default MINTEQA2 thermodynamic database (for details, see Section S3).

### 2.3 Hg(II) Reduction Experiments

All experiments were conducted in the absence of organic matter. Anoxic ultrapure water (UPW; resistivity >18.2 MΩ cm, TOC < 2 ppb, Milli-Q, Millipore) was prepared by boiling water and subsequent purging with N_2_ for at least 2 h during cooling before transfer into an anaerobic glovebox (4:96 H_2_/N_2_) for equilibration with the gas phase overnight. For all experiments, Hg(II) was added from a 1000 mg L^–1^ NIST-3133 solution in 10% (v/v) HNO_3_. Solution pH was buffered with 20 mM 3-morpholinopropane-1-sulfonic acid (MOPS) and adjusted to 8 using 6 M NaOH. For experiments investigating Hg(II) reduction by magnetite and Fe(II) bound to goethite, the pH was buffered with 20 mM 2-(N-morpholino)ethanesulfonic acid (MES) and adjusted to 6.5 to reduce the reaction rate by shifting the Hg(II) speciation to a lower proportion of Hg(OH)_2_.

The oxidizing trapping solution used to reoxidize and stabilize volatilized Hg(0) consisted of 40% (v/v) inverse aqua regia with a HNO_3_/HCl ratio of 3:1 (hereafter, iAR) with HCl replaced by 0.2 M BrCl solution^49^ (hereafter, BrCl) (for details, see Section S1). Glass frits leading to the traps were used to disperse the gas flow to increase the reaction surface area and trapping efficiency.

All experiments were prepared in triplicate in airtight glass serum bottles in an anaerobic glovebox. Serum bottles were wrapped with aluminum foil to prevent photochemical influences and sealed with bromobutyl stoppers and aluminum crimp caps to ensure oxygen-free conditions in all experiments conducted outside of the glovebox. The suitability of such stoppers for work with Hg(0) has been tested in previous studies.^18,50^ The serum bottles were then brought to a fume hood where purging with N_2_ (5.0 purity, plus in-line gold trap to minimize the Hg blank) was started immediately, and the experiments were initiated.

#### 2.3.1 Hg(II) Reduction by Dissolved and Surface-Bound Fe(II) in Open System Experiments

Goethite (BET surface area: 33.6 m^2^ g^–1^) and boehmite (179.8 m^2^ g^–1^) were weighed into serum bottles to reach 12 m^2^ L^–1^ and 4500 m^2^ L^–1^, respectively. The serum bottles were subsequently brought into the glovebox for equilibration with the atmosphere overnight. Multiple reactors were prepared in parallel by adding Hg(II) (1 μM) to the buffer solutions or buffer-mineral suspensions in the glovebox. Both, homogeneous and heterogeneous experiments were initiated by adding the Fe(II)-stock solution to the sealed reactors with a needle and syringe to reach a Fe(II) concentration of 12.5 μM. Reactors were continuously mixed by stirring with a magnetic stir bar and purged with N_2_ to effectively transfer the produced Hg(0) to the oxidizing traps. Reactors were sacrificed after each time step by quenching the reduction reaction with HCl (1 M). Purging was continued for 15 min to transfer the remaining dissolved Hg(0) to the oxidizing traps. For Fe(II)-goethite experiments, the HCl concentration was then increased to 6 M to dissolve the mineral and facilitate homogeneous and quantitative sampling. Reactor samples were then stabilized by adding 1% (v/v) BrCl. For Fe(II)-boehmite experiments, the remaining Hg in reactors was desorbed by increasing the HCl concentration to 6 M and adding 1% BrCl. Kinetic reduction experiments with dissolved Fe(II) were carried out at 0.5 and 10 mM Cl^–^ concentration, adjusted by changing the molarity of HCl in the diluted Fe(II) stock solution.

#### 2.3.2 Homogeneous Hg(II) reduction by Fe(II) in Closed System Experiment

The isotope exchange experiment was carried out similarly to the kinetic experiments at a 0.5 mM Cl^–^ concentration. The reactors were initially not purged to allow isotope exchange between the produced Hg(0) and Hg(II) in solution. After each time step, the reaction was quenched with HCl (1 M) and the reactors were purged for 30 min to transfer Hg(0) to the oxidizing traps.

#### 2.3.3 Hg(II) Reduction by Magnetite in Open System Experiments

Experiments were prepared from a stock suspension of magnetite (BET surface area: 40.8 m^2^ g^–1^) to reach ∼2 m^2^ L^–1^. To initiate the reaction, Hg(II)-stock was added via syringe and needle to reach a final concentration of 1 μM. Samples were homogenized by purging, and no stir bar was added to avoid magnetic adherence of magnetite particles. Samples were collected from the reactors via syringe and needle, and an aliquot was filtered (0.2 μm, cellulose acetate). The magnetite in withdrawn suspensions was subsequently dissolved by increasing the HCl concentration to 6 M. Traps were exchanged at every time point resulting in a time-integrated semi-instantaneous product from which the cumulative product was calculated.

#### 2.3.4 Control Experiment

A control experiment was carried out without the addition of any reducing agent at 0.5 mM Cl^–^ for 16 h. The stability of pH in reactors was measured in preliminary experiments before stabilization, and the change in pH was minor (±0.02, Section S1.2).

### 2.4 Hg Concentration and Isotope Analysis

Mercury concentrations were measured by using cold vapor atomic absorption/fluorescence spectrometry (CV-AAS/AFS; DMA-80L) after online reduction of Hg(II) to Hg(0) by stannous chloride (SnCl_2_). Samples were diluted using 1% (v/v) BrCl to 2.5–50 nM Hg and prereduced with 30% (w/v) hydroxylamine hydrochloride (NH_2_OH·HCl) immediately before concentration measurements. Procedural blanks consisted of 1% BrCl and the iAR trap solution diluted 1:10 with UPW. Repeated measurements of NIST-3133 were included as quality control throughout the measurements and recoveries were 98 ± 2% (*n* = 85).

Mercury isotope ratios were measured with multicollector inductively coupled plasma mass spectrometry (MC-ICPMS; Nu Plasma II) using a cold vapor generation system (HGX-200) for Hg introduction and a desolvating nebulizer for Tl introduction (Aridus II). Mass bias and instrumental drift were corrected with standard-sample bracketing by using NIST-3133 and Tl doping by using NIST-997. The accuracy and analytical precision of each session were determined with repeated measurements of the secondary standard “ETH Fluka” (Table S5). This method was previously described in detail,^51^ and further information, including the definition of δ- and Δ-values, is provided in the SI (Section S4).

### 2.5 Calculation of Enrichment Factors (ε, E)

Fractionation models were fitted using an Excel Solver routine, minimizing the sum of squared residuals between predicted and measured Hg isotope values of reactors and traps assuming a constant isotope enrichment factor (ε, E; see Section S10 for definitions) between the isotope ratios of Hg(II) and the instantaneously produced Hg(0). For the fitting of fractionation models, the value for fraction reacted (*f*) was calculated based on measured Hg concentrations in reactors (*f*_remaining_) because these values are considered more robust and reliable than values based on trap concentrations (*f*_reacted_) due to the higher risk of loss of volatile Hg(0). An isotope mass balance was calculated using *f*_remaining_ for each pair of reactor and trap and the respective isotope ratio (provided both were analyzed).

## 3 Results and Discussion

**Figure 1:**
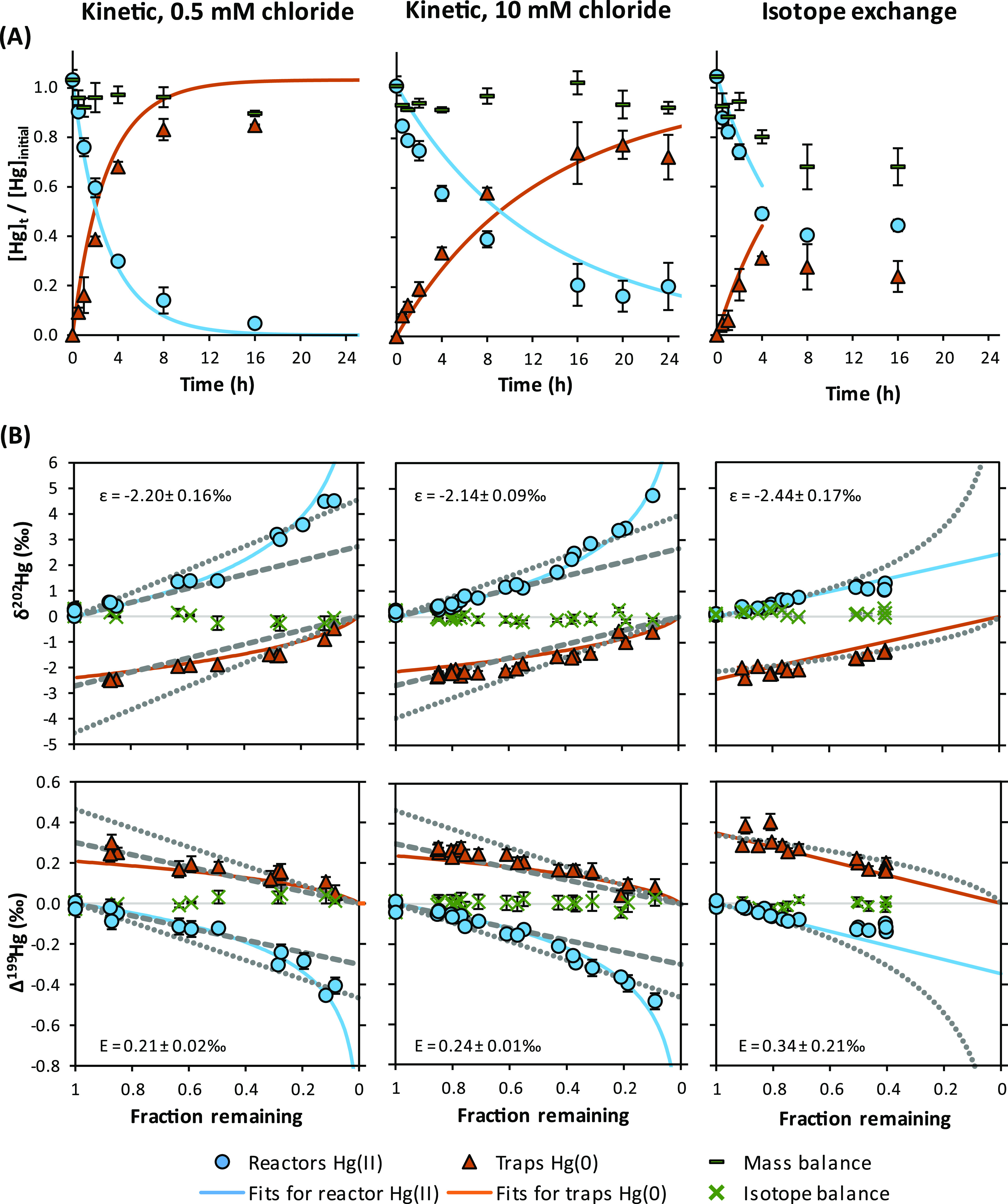
(A) Time–concentration plots for the reduction of Hg(II) by dissolved Fe(II). Progress of reaction is reported as averages of individual replicate reactors and traps (error bars = 1SD). Solid lines represent model fits based on the calculated rate constants. (B) Isotope ratios of individual reactors and traps for mass-dependent (δ^202^Hg, MDF) (Error bars are smaller than the data symbols) and mass-independent fractionation (Δ^199^Hg, MIF). Solid lines represent best fits for reactants and cumulative products, gray lines are for comparison of linear models for the open systems based on reactors (dotted) or traps (dashed), and a Rayleigh model for the closed system (isotope exchange experiment). Further information on the determined enrichment factors and their uncertainties using different fitting approaches can be found in Section S11.

**Figure 2:**
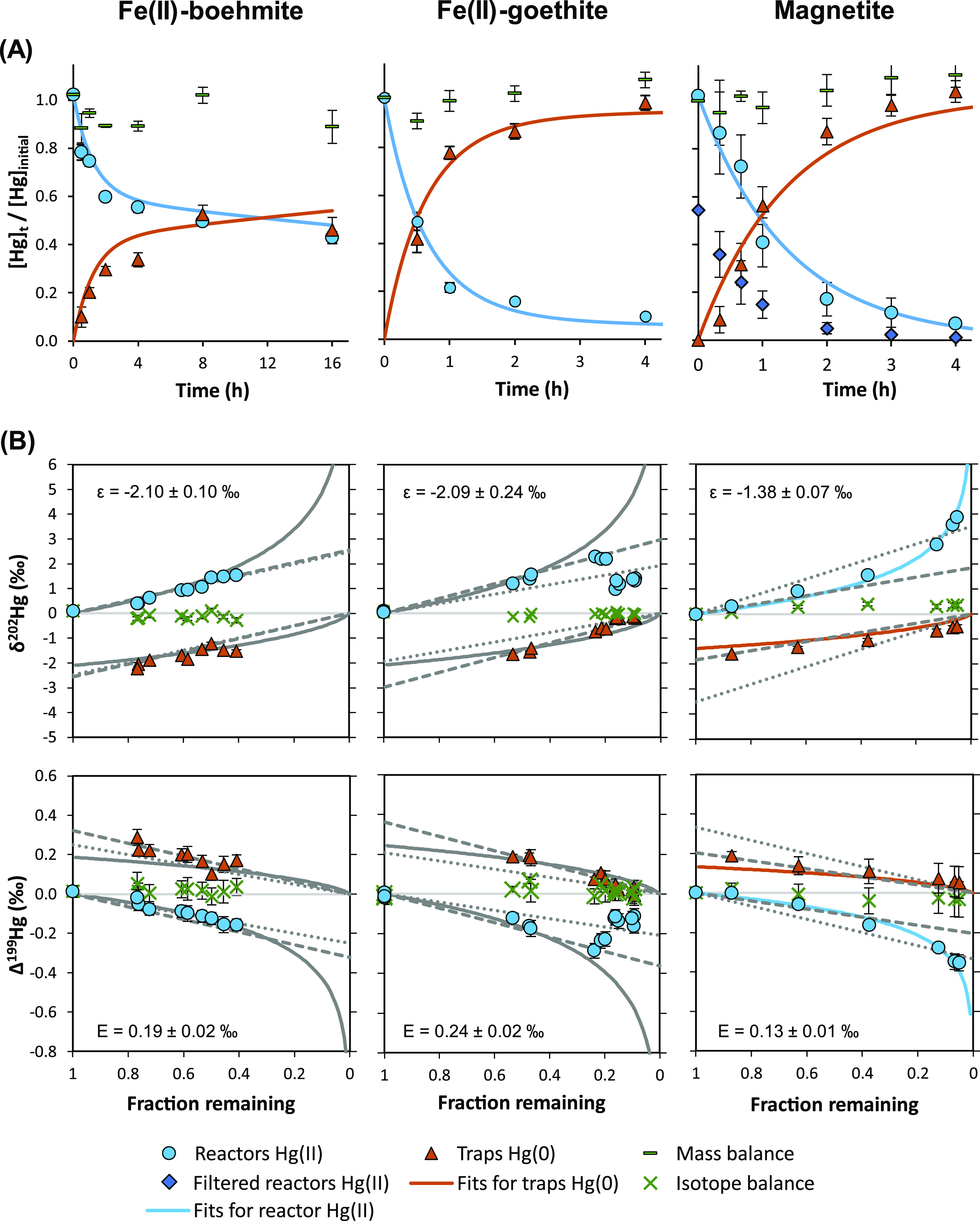
(A) Time–concentration plots for heterogeneous reduction of Hg(II) by Fe(II)-boehmite, Fe(II)-goethite, and magnetite in open systems. Progress of reaction is reported as averages of individual replicate reactors and traps (error bars = 1SD). Solid lines represent model fits based on the calculated rate constants. (B) Isotope ratios of individual reactors and traps for mass-dependent (δ^202^Hg, MDF) (Error bars are smaller than the data symbols) and mass-independent fractionation (Δ^199^Hg, MIF). Rayleigh model fits for reactants and cumulative products are represented by solid lines, linear equilibrium model fits based on reactors are represented by dotted lines, and fits based on traps are represented by dashed lines, respectively. Further information on the determined enrichment factors and their uncertainties using different fitting approaches can be found in Section S11.

The Hg(II) concentrations in the reactors of all experiments decreased rapidly after mixing with Fe(II) or magnetite (Figures 1 and 2). In control runs without reducing agent 14 ± 1% (*n* = 3) of Hg(II) were lost within 16 h and recovered in the traps, similar to comparable control experiments for Hg loss in UPW.^15,23^

### 3.1 Homogeneous Reduction of Hg(II) by Fe(II)

The kinetics of Hg(II) reduction by dissolved Fe(II) were previously described to follow a second-order rate law with a strong pH dependence.^14^
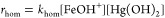
1In our study, the overall second-order rate constant (*k*_hom_) for the homogeneous experiments was −1.88 ± 0.14 × 10^4^ M^–1^ min^–1^, similar to the previously reported rate constant of −7.19 × 10^3^ M^–1^ min^–1^.^14^ The increase in Cl^–^ concentration from 0.5 to 10 mM slowed the overall reduction rate by shifting the aqueous Hg species distribution toward a higher proportion of stable chloro-complexes (HgClOH, HgCl^+^, HgCl_2_)^52^ and lowering the concentration of the otherwise dominant Hg(OH)_2_ species at the chosen experimental conditions (pH 8). Both experiments could therefore be fitted using the same rate law and rate constant, indicating that the chloro-complexes are nonreactive on the time scales of the overall reduction reaction. A detailed derivation of rate constants is provided in Section S7.

#### 3.1.1 Isotope Exchange Effect

For the isotope exchange experiment, the rate constant was calculated for the initial time points (up to 4 h; *k* = −7.6 ± 1.9 × 10^3^ M^–1^ min^–1^) indicating slower kinetics compared to the kinetic experiment, suggesting that a back-reaction of Hg(0) to Hg(II) starts to occur already in the initial phase of the experiment.

#### 3.1.2 Isotope Fractionation in Homogeneous Experiments

The reduction of Hg(II) resulted in MDF of both the remaining Hg(II) and the produced Hg(0) pool. The remaining Hg(II) became increasingly enriched in heavy isotopes compared to the initially added NIST-3133 (δ^202^Hg = 0‰). In all experiments, MIF of odd-mass isotopes (^199^Hg and ^201^Hg) was observed (Figures 1 and 2, Section S9). Additionally, MIF of even-mass isotopes was detectable in all experiments for ^200^Hg except the magnetite experiment and for ^204^Hg in homogeneous experiments (Section S11). A Wilcoxon signed rank test was used to test if odd- and even-mass isotope ratios in reactors were statistically different from trap values (Section S12). The extent of MIF was larger in experiments with larger MDF, as expected for systems influenced by the NVE (Section 3.4).

While the open system experiments followed the Rayleigh fractionation model, the results of the isotope exchange experiments were best described by a linear equilibrium fractionation model (Figure 1). Usually, isotope exchange is fast if only one electron transfer is involved with no change of coordination, such as in the case of Fe(II) and Fe(III) isotope exchange.^53^ For systems involving the transfer of several electrons and a coordination change the exchange kinetics are much slower, such as for the reduction of uranium(VI)^54^ and chromium(VI).^55^ Despite isotope exchange for Hg consisting of the exchange of two electrons, a rapid isotope exchange within minutes has been reported to overprint kinetic isotope effects during the reduction of Hg(II) by siderite and green rust.^18,56^ A similar effect is observed for the homogeneous reduction of Hg(II) by dissolved Fe(II) in our study, where equilibration occurred within the short time frame of the experiments (up to several hours). The formation of an Hg(I)-dimer as an intermediate species via collision of Hg(0) and Hg(II) was proposed as a likely mechanism responsible for a prolonged interaction and thus the rapid equilibration between Hg(0) and Hg(II).^18^

The lower recoveries in later time points in the isotope exchange experiments likely resulted from an accumulation of dissolved Hg(0) in the reactors and an incomplete transfer during the purging with subsequent loss of Hg(0) during the transfer of reactor samples before stabilization with BrCl. This leads to a larger uncertainty in the determination of *f*_reacted_. It has been demonstrated that the volatilization of Hg(0) from solution into the gas phase can result in kinetic Hg isotope fractionation.^57^ However, it is unclear whether active purging will lead to a similar effect. As a quality control, isotope balances were calculated for all measured pairs of reactors and traps (Figures 1 and 2, Tables S8.1–S8.7). The closed isotope balances in all of our experiments indicate that there was no loss of Hg resulting in an isotope fractionation artifact.

### 3.2 Heterogeneous Reduction of Hg(II)

#### 3.2.1 Hg(II) Reduction by Fe(II) Bound to Goethite and Boehmite

Compared to the homogeneous experiments, the Hg(0) production rate was lower in the presence of boehmite, but higher in the presence of goethite, which agrees well with published data.^14^ Observed rate constants for surface-catalyzed experiments (*k*_het_) were obtained based on the second-order reaction expression.^14^
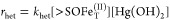
2where >SOFe_T_^(II)^ is the total sorbed Fe(II) (100% of total Fe in case of Fe(II)-boehmite at pH 8 and 25% of total Fe in case of Fe(II)-goethite at pH 6.5). The rate constants for heterogeneous experiments were calculated based on the parameters reported in Amirbahman et al.^14^ for the sorption of Fe(II) and Hg(II) to goethite and boehmite. The determined rate constants (*k*_Fe(II)-boehmite_ = −5.5 × 10^2^; *k*_Fe(II)-goethite-pH6.5_ = −6.6 × 10^4^) are in the same range as reported rate constants (*k*_Fe(II)-boehmite_ = −1.08 × 10^2^ M^–1^ min^–1^ and *k*_Fe(II)-goethite_ = −4.96 × 10^3^ M^–1^ min^–1^).^14^

#### 3.2.2 Hg(II) Reduction by Magnetite

The reduction of Hg(II) by Fe(II)-bearing minerals was suggested to follow pseudo-first-order kinetics because the Fe(II) surface site concentration is in large excess compared to Hg(II) concentrations.^15,21,23,27^
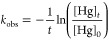
3For the magnetite experiment, an observed pseudo-first-order rate constant (*k*_obs_) of 11.9 ± 0.6 × 10^–3^ min^–1^ was determined and normalized to Fe(II) surface site concentration (*k*_s_ = 0.2 L μmol^–1^ min^–1^) for comparison with previous studies, using the specific surface area of the biogenically synthesized magnetite and published data of Fe(II) surface site density^58^ (Table S7.2). Both *k*_obs_ and *k*_s_ are very similar compared to values reported in previous experiments with magnetite at a comparable pH.^23^

#### 3.2.3 Isotope Fractionation in Heterogeneous Experiments

Isotope fractionation during Hg(II) reduction by magnetite followed a Rayleigh distillation model. However, this was not the case for Hg(II) reduction with Fe(II) in the presence of mineral surfaces. While the reaction progress was insufficient in the Fe(II)-boehmite experiment to clearly assign a fractionation model, a deviation from fractionation models was apparent for the Fe(II)-goethite experiments. The fractionation trend was initially similar to the homogeneous experiments, but the observed isotope ratios deviated from both Rayleigh and equilibrium models at later time points. While the reactor values were less enriched in heavy isotopes for reactors sacrificed at later time points, the traps mirrored this trend, resulting in a closed isotope balance.

This observed isotope fractionation trend in reactors sacrificed after longer reaction times in Fe(II)-goethite experiments is hypothesized to be influenced by the sorption of Hg(II) to the mineral surface or the interaction of Fe(II) with the goethite mineral surface. Experiments describing the exchange kinetics between Hg(II) and goethite reported the formation of a rapidly sorbed Hg pool, a pool with slower exchange kinetics, and the formation of a “non-exchangeable” fraction.^59^ The different equilibration times before initiating the reaction by adding Fe(II) may have led to differences in the sorbed Hg pools and affected Hg(II) reduction. The exposure of goethite to aqueous Fe(II) leads to rapid Fe atom exchange between solid-phase Fe(III) and aqueous Fe(II).^60^ Such Fe(II)-catalyzed recrystallization processes can affect the redox cycling of trace elements by structural incorporation and release.^60−63^ The formation of sorbed Hg(II) pools with different exchange kinetics and potential structural incorporation of Hg during Fe(II)-catalyzed recrystallization of goethite are expected to have influenced the isotope fractionation behavior. Although this was not apparent from the kinetics of Hg(II) reduction in the Fe(II)-goethite experiments, these processes could explain the observed fractionation trend that deviates from Rayleigh or equilibrium models exhibiting a smaller extent of isotope fractionation in later time points.

In the magnetite experiments, Hg(II) also gets partially adsorbed to the mineral surface, as can be seen from the difference between filtered and unfiltered reactor samples. Nonetheless, the isotope fractionation trend for Hg(II) reduction by magnetite was described well by Rayleigh fractionation and is distinct from the other experiments by exhibiting a lower ε.

### 3.3 Extent of Mass-Dependent Fractionation (MDF)

Small variations in the determined ε values can result from the chosen method of fitting a model and deriving ε (Section S10). While some studies use the linearized version of Rayleigh plots based on reactor isotope and concentration data to determine ε,^33^ the reported values in this study include the sum of squared residuals for fits using measured isotope ratios of both traps and reactor values. All fractionation factors calculated depend on a precise determination of the fraction reacted, and small errors in *f*_reacted_ can have a considerable influence on the model fit because of the logarithmic nature of the Rayleigh model. In addition to Rayleigh models, linear equilibrium models were fitted to kinetic experiments. For the homogeneous open system and magnetite experiments, the linear equilibrium models did not produce a satisfactory fit, resulting in a much larger sum of squared residuals. This suggests that the observed isotope fractionation is indeed kinetically driven. A comparison of ε values based on different fitting approaches is provided in Table S11.

The extent of the observed MDF was very similar for experiments with dissolved and surface-bound Fe(II) (ε^202^Hg between −2.20 and −2.09‰). This extent is slightly larger than that of the MDF reported for other Hg(II) reduction pathways (Table 1). The Hg(II) reduction by magnetite on the other hand showed a considerably lower extent of MDF (ε^202^Hg of −1.38‰, Table 1) which is also lower compared to the extent of MDF reported for siderite (−2.43 ± 0.38‰).^18^ In comparison to our magnetite experiments the reported siderite experiments were however conducted in a closed system and the initial kinetic effect was overprinted by isotope exchange.^18^ The closed system experiments with Fe(II) resulted in a very similar ε of −2.44 ± 0.17‰, suggesting a similar mechanism of isotope exchange. The results of the equilibrium model fit for MDF suggest an ε^202^Hg in the same range as computationally predicted values for dissolved Hg(OH)_2_ relative to elemental Hg vapor (−2.19^51^ to −2.44‰^64^).

**Table 1:** Compilation of the Isotope Enrichment Factors for Hg(II) Reduction in Homogeneous and Heterogeneous Systems and Theoretical Predictions^a^

experiment/reducing agent	ε^202^Hg (‰)	E^199^Hg (‰)	E^200^Hg (‰)	E^201^Hg (‰)	E^204^Hg (‰)	Δ^199^Hg/Δ^201^Hg	Δ^200^Hg/Δ^201^Hg	Δ^204^Hg/Δ^201^Hg
This Study:								
Fe(II), open system, 0.5 mM Cl^–^	–2.20 ± 0.16	0.21 ± 0.02	0.02 ± 0.003	0.13 ± 0.01	–0.02 ± 0.01	1.58 ± 0.08	0.26 ± 0.04	–0.15 ± 0.07
Fe(II), open system, 10 mM Cl^–^	–2.14 ± 0.09	0.24 ± 0.01	0.03 ± 0.003	0.15 ± 0.01	–0.03 ± 0.005	1.62 ± 0.07	0.23 ± 0.03	–0.23 ± 0.06
Fe(II), closed system, 0.5 mM Cl^–^	–2.44 ± 0.17	0.34 ± 0.02	0.04 ± 0.01	0.21 ± 0.02	–0.05 ± 0.01	1.60 ± 0.05	0.17 ± 0.03	–0.20 ± 0.07
Fe(II)-boehmite, open system	–2.10 ± 0.10	0.19 ± 0.02		0.14 ± 0.02		1.57 ± 0.10	0.24 ± 0.05	
Fe(II)-goethite, open system (pH 6.5)	–2.09 ± 0.12	0.24 ± 0.02		0.16 ± 0.03		1.56 ± 0.12	0.21 ± 0.05	
magnetite, open system	–1.38 ± 0.07	0.13 ± 0.01		0.09 ± 0.01		1.59 ± 0.09		
Comparison to Other Hg(II) Reduction Pathways:								
SnCl_2_ (trial 1/trial 2)	–1.56 ± 0.11/–1.77 ± 0.11	0.17/0.26		0.11/0.17		1.59/1.62		
dissolved organic matter^41^	–1.52 ± 0.06	0.19		0.12		1.60 ± 0.12		
microbial reduction^33−35,80^	–0.60 to −1.8	no MIF		no MIF		no MIF		
organically mediated photoreduction^36,37^	–0.60 to −1.09	(−)MIF		(−)MIF		1.0 to 1.31		
photoreduction by sulfurless/S-containing ligands^81^	–1.71 ± 0.03 (serine)/–1.32 ± 0.07 (cysteine)	(−)MIF/(+)MIF		(−)MIF/(+)MIF		1.1 to 1.67		
photoreduction of intracellular Hg(II)^82^	–0.7 to −0.8	0.89 to 1.03				≈1.0		
Closed System Hg(II) Reduction Experiments:								
Hg(II) reduction by siderite^18^	–2.43 ± 0.38^c^	0.28 ± 0.06		0.27 ± 0.14		1.06^b^		
Hg(II) reduction by green rust^18^	–2.28 ± 0.40^c^	0.28 ± 0.06		0.27 ± 0.14		1.06^b^		
Hg(II)–Hg(0) equilibration^18^ (No Cl^–^/10 mM Cl^–^)	–2.63 ± 0.37^c^/–2.77 ± 0.70^c^	0.28 ± 0.21^c^		0.20 ± 0.12^c^		1.44		
Theoretical Predictions for Equilibrium Fractionation of Relevant Hg(II) Species Relative to Hg(0) Vapor:								
Hg(OH)_2_	–2.19^51^ to −2.44^64^	0.20^51^ to 0.23^64^	0.03^51^ to 0.04^64^	0.12^51^ to 0.14^64^	–0.04	1.65^51^	0.24	–0.29
HgClOH	–2.17^51^	0.22^51^	0.03^51^	0.13^51^	–0.04	1.65^51^	0.24	–0.29
HgCl_2_	–2.09^51^	0.25^51^ to 0.29^64^	0.04^51^ to 0.05^64^	0.15^51^ to 0.18^64^	–0.04	1.65^51^	0.24	–0.29

a Errors for ε and E are reported as 2SE of regressions.

b The uncertainty of Δ^201^Hg data precludes a confident comparison.

c uncertainties reported as 2SD

Variation in the magnitude of isotope fractionation was previously explained by the reaction rate or rate constants (*k*) in uranium,^65^ chromium,^66−69^ zinc,^70^ and iron^71^ isotope systems. Hydrolysis and ligation of Fe(II) change the reduction potential (*E*°) of the Fe(II)–Fe(III) half-reaction and lead to large differences in reduction rates.^68,72,73^ Although a universal relationship between the thermodynamic driving force of redox reactions and the reaction rate does not exist,^74^ linear free energy relationships (LFERs) were observed for such reactions. The logarithms of rate constants were described as a function of the free energy of the reaction (Δ*G*_r_°), resulting in a linear relationship between redox-driven isotope fractionation factors (ε) and Δ*G*_r_° of the electron transfer.^68^ Rather than the observed reaction rates, differences in *E*° can, therefore, be considered as the major driving force of variability in ε. Observed reaction rates in turn are also dependent on *E*° leading to an apparent correlation between ε and *k*_obs_.^68^ In our experiments, there is no observable difference in MDF or MIF in experiments with a higher Cl^–^ concentration, despite the slower reduction rate. In both cases, the redox active species are assumed to be the same (Hg(OH)_2_ and FeOH^+^) and the slower reaction in the Cl^–^ experiment resulted from lower Hg(OH)_2_ concentrations due to a shift in Hg speciation (Section S3). These similar fractionation factors are in good agreement with the finding that both experiments could be fitted with the same second-order rate constant. The addition of Cl^–^ further only has a minor effect on the Fe speciation (Section S3), and therefore the *E*° of the Fe(II)–Fe(III) half-reaction remains practically unchanged.

While the reactivity of Fe(II)-complexes in solution can be predicted based on LFERs and the reduction potential of the Fe(II)-complexes, predictions of the reactivity of Fe(II) bound to surfaces are difficult because the reduction potential of such Fe(II) species is usually not known.^75^ The similarity in rate constants among Hg(II) reduction experiments in homogeneous and goethite-catalyzed systems was previously suggested to result from a similarity in reduction potentials between adsorbed Fe(II) and the FeOH^2+^/FeOH^+^ couple.^14^ We observed a similar extent of Hg isotope fractionation in experiments with dissolved Fe(II) and surface-bound Fe(II), which could be explained by such a similarity in the reduction potentials. Predicting the redox behavior of magnetite is challenging^76^ and reported measured redox potentials for magnetite range over a wide range (at neutral pH from +0.66^77^ to −0.38 V^78^), which can be mainly attributed to differences in the magnetite stoichiometry (Fe(II):Fe(III) ratio).^79^ Whether the observed difference in the magnitude of the Hg isotope fractionation between magnetite and Fe(II)-mediated reduction of Hg(II) is a result of different thermodynamic driving forces remains an open question, but we suggest that it might be a plausible explanation based on our data.

### 3.4 Mass-Independent Fractionation Effects

Reduction of Hg(II) was previously reported to cause MIF.^18,33,34,36,37,41,82^ The two plausible mechanisms explaining MIF in the Hg isotope system are the nuclear volume effect (NVE) and the magnetic isotope effect (MIE) (Section S9). The observed E-values for ^199^Hg, ^200^Hg, ^201^Hg, and ^204^Hg in our experiments agree well with the theoretically predicted NVE values for equilibrium fractionation of relevant Hg(II) species relative to Hg(0) vapor (Table 1). Despite these predictions being based on theoretical equilibrium fractionation and the lack of theoretical calculations for NVE in kinetic reactions to date,^83,84^ we also observed similar NVE-related MIF for kinetic Hg isotope fractionation.

The ratio (“slope”) of Δ^199^Hg and Δ^201^Hg is useful to differentiate between MIE and NVE as a driver for MIF. The exact controls of Δ^199^Hg/Δ^201^Hg ratios during MIE are not clearly understood, but observed ratios range from ∼1 to 1.4 for experimental photochemical transformations.^36,37^ For NVE the theoretically predicted slope is 1.65^51^ and experimentally observed ratios for dark abiotic reduction and equilibrium isotope fractionation are in close agreement with the theoretical prediction.^41,51,85^ No MIF was reported for microbial reduction of Hg(II).^35,80^ In all of our experiments odd-mass MIF and Δ^200^Hg were always opposite in sign compared to MDF while Δ^204^Hg had the same sign as MDF, which is characteristic of the MIF caused by NVE (Section S9).^64^ The Δ^199^Hg/Δ^201^Hg slope was between 1.56 and 1.62 indicating that the observed MIF was caused by the NVE (Table 1).

**Figure 3:**
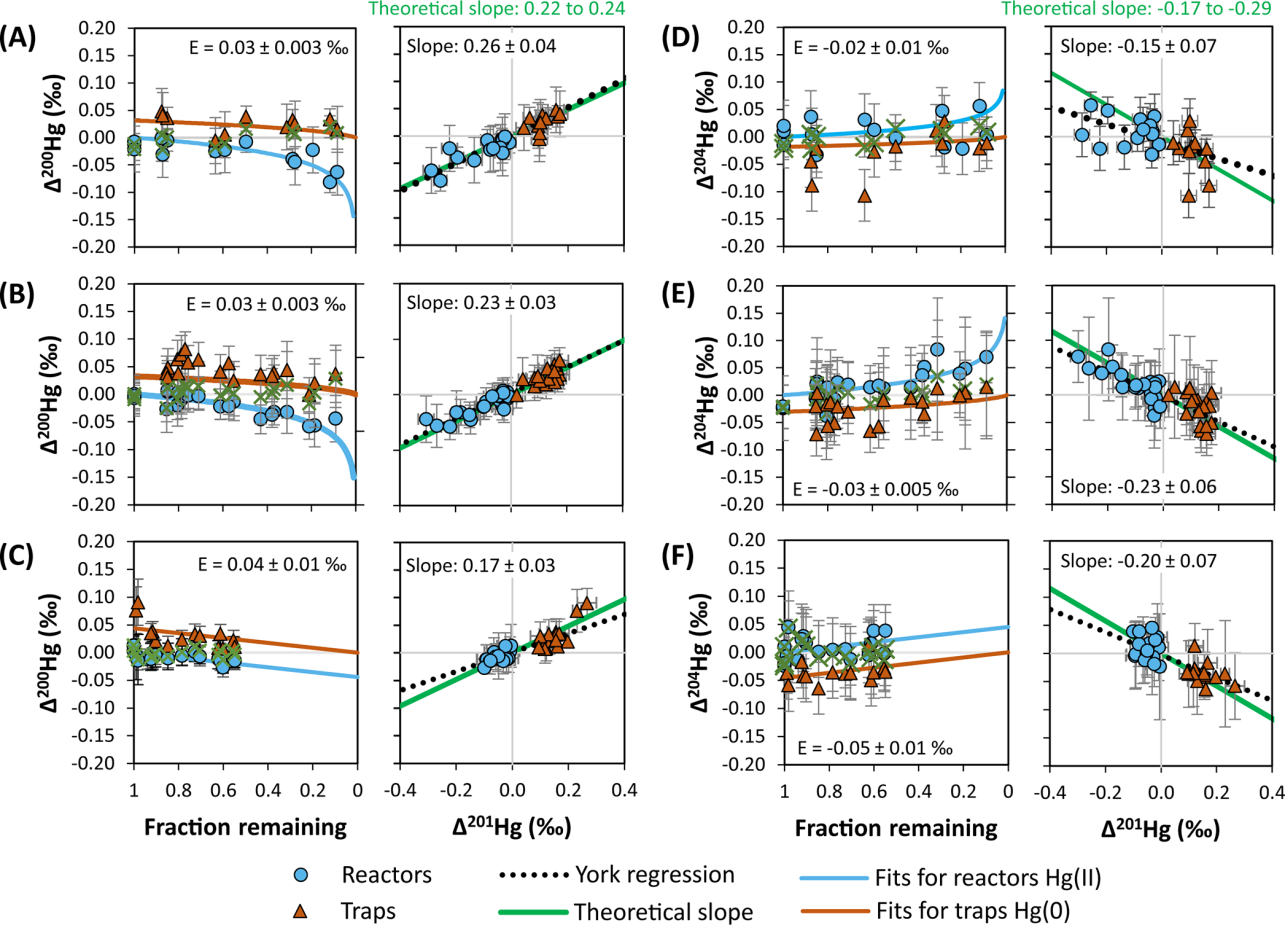
Mass-independent fractionation of ^200^Hg for (A) kinetic experiments at 0.5 mM chloride and (B) kinetic experiments at 10 mM Cl^–^ and (C) isotope exchange experiments. Mass-independent fractionation of ^204^Hg in (D) kinetic experiments at 0.5 mM chloride, (E) kinetic experiments at 10 mM Cl^–^, and (F) isotope exchange experiments.

Based on the anomalies of nuclear charge radii^86−88^ and scaling factors for Hg isotopes relative to ^202/198^Hg^51^ we calculated theoretical slopes for Δ^200^Hg/Δ^201^Hg (0.22 to 0.24) and Δ^204^Hg/Δ^201^Hg (−0.17 to −0.29). The observed slopes between 0.17 and 0.26 for Δ^200^Hg/Δ^201^Hg in our homogeneous experiments agree with these theoretical predictions (Figure 3, Table 1). For Δ^204^Hg/Δ^201^Hg, the lower abundance of ^204^Hg results in lower signal intensities during the isotope analysis and, therefore, a larger uncertainty. Additionally, there is a larger range in the predicted slope resulting from differences in reported nuclear charge radii for ^204^Hg (Table S9.3).^86−88^ The observed slopes are nonetheless in good agreement with the theoretical predictions, and results of the Wilcoxon signed rank test demonstrate a significant difference between isotope ratios in reactors and traps in the homogeneous experiments. The even-MIF results of the heterogeneous experiments were less clear, but still mostly consistent with NVE predictions (Figure S9.3).

### 3.5 Environmental Implications

Reduction of Hg(II) to Hg(0) plays a key role in the geochemical cycle, and Hg redox transformations significantly alter its fate in the environment. The goal of applying Hg isotope ratio measurements to trace transformation processes in the environment and understanding their impacts on Hg isotope systematics of Hg atmosphere-surface exchange requires a precise and well-defined knowledge of the fractionation trends arising from these processes.

The results of this study suggest that the isotope fractionation resulting from Hg(II) reduction is complex, and extrapolating these experimental findings to environmental conditions requires careful consideration of effects such as overprinting by secondary processes. In closed systems allowing for equilibration between Hg(II) and Hg(0), the initial kinetic isotope effects are rapidly overprinted by the isotope exchange. Such an overprinting of kinetic isotope fractionation can be expected in many geochemical settings, such as groundwater aquifers or surface waters, where Hg redox processes occur, and Hg(II) and Hg(0) coexist, and it needs to be considered when isotope signatures are used to trace Hg in environmental systems. With the presence of mineral surfaces and the increasing complexity of the system, the interpretation of isotope ratios regarding process tracing becomes even more challenging. Due to the high abundance of Fe and Fe-minerals in natural systems, adsorbed and structural Fe(II) are expected to have a large effect on the observed Hg isotope fractionation.

Linear free energy relationships predict an effect of ligands (e.g., chloride and dissolved organic carbon) on the extent of Hg isotope fractionation. However, when the kinetic reaction rate is dominated by one species, the presence of other ligated species with a much slower reaction rate has a minor effect on the extent of isotope fractionation, as shown by the similar isotope ε in experiments with higher Cl^–^ concentrations compared to experiments with lower Cl^–^ concentrations.

This study represents an important addition to the database of Hg isotope enrichment factors for individual processes. Our results show that Hg(II) reduction by dissolved Fe(II) in open systems leads to kinetic MDF with an ε that is larger compared to other abiotic reduction pathways and MIF that is distinct from other reduction pathways. By combining MDF and MIF, dark abiotic Hg(II) reduction can be distinguished from photochemical or microbial Hg(II) reduction pathways, demonstrating that multidimensional Hg isotope signatures can be a powerful tool for process tracing. Additionally, we report experimental evidence for MIF of even-mass Hg isotopes related to NVE, consistent with theoretical predictions based on nonlinearity of nuclear charge radii. Despite the small magnitude of the documented even-mass MIF caused by the NVE, we propose that it may need to be considered in the interpretation of small even-mass MIF signal found in environmental samples, which is often assumed to be generated exclusively by atmospheric processes.^89,90^

## Supporting Information Available

Additional experimental details and further description of the experimental setup (Text S1); mineral synthesis protocols and mineral characterization (Text S2 and Figure S2); description of modeling of Hg and Fe species at initial conditions and reporting of used equilibrium constants (Text S3, Figures S3.1–S3.3, and Table S3); additional details about the Hg isotope analysis procedure (Text S4); assessment of accuracy and precision of isotope analysis (Table S5); reporting of results of sorption experiments (Figure S6); discussion of reduction kinetics and calculation of rate constants (Text S7; Tables S7.1–S7.4 and Figure S7); reporting of isotope ratios of individual samples (Tables S8.1–S8.7); reporting of additional results of odd- and even-mass MIF (Text S9, Figures S9.1–S9.6; Tables S9.1–S9.5); discussion of different method approaches for the calculation of enrichment factors (Text S10); comparison of enrichment factors derived from different approaches (Table S11); and results of paired, two-sided Wilcoxon signed rank test to test for significant differences between measured reactor and trap isotope ratios for Δ^199^Hg, Δ^200^Hg, Δ^201^Hg, and Δ^204^Hg of each experiment (Table S12) (PDF)
