# Supplementary material for: Mercury Isotope Fractionation during Dark Abiotic Reduction of Hg(II) by Dissolved, Surface-Bound, and Structural Fe(II)
Source: Environ Sci Technol. 2023 Sep 25;57(40):15243–54. doi: 10.1021/acs.est.3c03703 (PMC10569049; doi:10.1021/acs.est.3c03703)
Supplement: Supplementary file 1 — es3c03703_si_001.pdf [file es3c03703_si_001.pdf]

Supporting information (SI) for:

Mercury isotope fractionation during dark abiotic reduction of Hg(II) by dissolved, surface-bound, and structural Fe(II)

Lorenz Schwab[1,2,3]*, Niklas Gallati[1], Sofie M. Reiter[1], Richard L. Kimber[1], Naresh Kumar[1,4], David S. McLagan[5,6,7], Harald Biester[5], Stephan M. Kraemer[1], Jan G. Wiederhold[1*‡]

[1]Department of Environmental Geosciences, Centre for Microbiology and Environmental Systems Science, University of Vienna, Josef-Holaubek-Platz 2, 1090 Vienna, Austria

[2]Doctoral School in Microbiology and Environmental Science, University of Vienna, 1030 Vienna, Austria

[3]Environmental Engineering Institute IIE-ENAC, Soil Biogeochemistry Laboratory, École Polytechnique Fédérale de Lausanne (EPFL), Route des Ronquos 86, 1951 Sion, Switzerland

[4]Soil Chemistry and Chemical Soil Quality Group, Department of Environmental Sciences, University of Wageningen, Droevendaalsesteeg 3a, 6708 Wageningen, Netherlands

[5]Environmental Geochemistry Group, Institute of Geoecology, Technische Universität Braunschweig, Langer Kamp 19c, 38106 Braunschweig, Germany

[6]Department of Geological Sciences and Geological Engineering, Queen’s University, Kingston, Ontario, K7L 3N6, Canada

[7]School of Environmental Studies, Queen’s University, Kingston, Ontario, K7L 3N6, Canada

‡Present Address: Federal Institute of Hydrology (BfG), Department G – Qualitative Hydrology, Am Mainzer Tor 1, 56068 Koblenz, Germany

This file contains 44 pages, 21 tables, and 11 figures.

Table of Contents

S1 Materials and methods S2

S1.1 Glove box conditions S2

S1.2 Experimental setup and conditions S2

S1.3 Fe(II)-stock solution S2

S2 Mineral synthesis and characterization S3

S2.1 Boehmite (γ-AlOOH) S3

S2.2 Goethite (α-FeOOH) S3

S2.3 Biogenic magnetite (Fe_3O_4) S3

S3 Modeling of Hg and Fe species at initial conditions S6

S4 Hg isotope analysis S9

S5 Assessment of accuracy and precision of isotope analysis S10

S6 Sorption experiments S11

S7 Reduction kinetics and calculation of rate constants S12

S8 Isotope ratios of individual samples S19

S9 Odd and even mass MIF S30

S10 Calculation of enrichment factors (ε, E) S38

S11 Comparison of enrichment factors derived from different model approaches S39

S12 Statistical testing S40

References S41

S1 Materials and methods

All glassware was acid washed in 1.4 M HNO_3 for at least 24 h and rinsed with ultra-pure water (UPW; resistivity >18.2 MΩ cm, TOC< 2 ppb, Milli-Q, Millipore). For stabilization of Hg in solution a 0.2 M BrCl solution (hereafter, BrCl) was prepared following the protocol reported in Bloom et al.[1] The reagents (KBr and $KBrO_3$) used for the preparation of the BrCl solution were heated to 220 °C for 24 h before use to remove any potential mercury contamination. The solution was stored at 4 °C. The 1 % BrCl solution used as sample and standard matrix was prepared by dilution of the BrCl solution in UPW.

Samples for Hg concentration analysis were pre-reduced with $NH_2OH{\cdot}HCl$ to destroy the free halogens according to EPA method 1631.[2] Stannous chloride ($SnCl_2{\cdot}2H_2O$, ≥98%, AnalaR NORMAPUR®, < 0.05 ppm Hg, VWR chemicals) was purged with N_2 gas for half an hour to remove traces of Hg prior to its use for Hg concentration or isotope analysis.

S1.1 Glove box conditions

All preparation steps that involved oxygen-sensitive Fe(II) or Fe(II)-minerals were conducted in an oxygen-free glovebox (Toepffer Lab Systems). The atmosphere in the glovebox was kept at 96 % nitrogen (N_2) and 4 % hydrogen (H_2) by an automatic gas controller. Oxygen-free conditions were strictly maintained (O_2 < 1 ppm) and O_2 and H_2 were continuously monitored.

S1.2 Experimental setup and conditions

MOPS buffer (3-morpholinopropane-1-sulfonic acid) was chosen as a non-complexing buffer for all experiments at pH 8[3–5]. For experiments at pH 6.5 MES buffer was used (2-(N-morpholino)-ethanesulfonic acid). In many laboratory studies, including experiments investigating the reduction of Hg(II) by Fe(II) or Fe(II)-minerals, MOPS[6–8] and MES[8,9] buffers have been used as inert buffers. Solution pH was measured in replicate reactors of preliminary experiments before stabilization with HCl. In these experiments the reactors were sampled continuously at each timestep and not sacrificed. Solution pH after the experiments was 8.04 ± 0.02 (mean ± SD).

S1.3 Fe(II)-stock solution

A Fe(II) stock solution was prepared following the description of Dideriksen et al.[10] by dissolving $Fe(II)Cl_2{\cdot}4H_2O$ in oxygen-free water inside the glovebox to a concentration of 1.5 M. The pH of the solution was increased to pH 6 by dropwise addition of 6 M NaOH in order to precipitate Fe(III)-impurities as a green slurry. After centrifugation at 6000 rcf for 5 min the supernatant was filtered through a 0.2 µm cellulose acetate filter and stabilized by acidification with 6 M HCl to decrease pH <3. The Fe(II)-stock solution was stored in the dark in the anaerobic glove box and the Fe(II) and total Fe concentration determined before use by colorimetry, using the ferrozine assay.[11,12] For total Fe concentrations, aliquots were reduced with 10 % (w/v) $NH_2OH{\cdot}HCl$ dissolved in 1 M HCl. Samples for Fe(II) and total Fe were mixed with a 0.1 % (w/v) solution of ferrozine in 50 % (w/v) ammonium acetate buffer. Absorbance was measured at 562 nm in microplates with a plate reader (Tecan Infinite 200 Pro).

S2 Mineral synthesis and characterization

The specific surface area of the minerals was obtained from Brunauer-Emmett-Teller (BET) analysis using N_2 physisorption isotherms after overnight degassing at 105 °C (Quantachrome 95 Nova 2000e). X-ray powder diffraction (XRD) was used for characterization of the freshly synthesized minerals (Rigaku Miniflex 600). Phase identification / confirmation was performed in Smart Lab Studio II software using the Crystallography Open Database (COD) reference patterns. Magnetite and siderite were loaded onto an air-sensitive sample holder enclosed with an air-tight Kapton dome inside the oxygen-free glove box and measured immediately after transferring the sample holder to the instrument. The dome was kept closed over the sample throughout the analysis.

S2.1 Boehmite (γ-AlOOH)

CATAPAL D® high purity alumina (boehmite) was purchased from Sasol Chemicals. This material was well described in previous studies.[13–15] The measured mineral surface area of 179.8 m^2 g^{-1} agreed with reported values in the literature of 180 m^2 g^{-1}. The XRD pattern aligned well with the reference pattern for boehmite in the COD database (Figure S2A).

S2.2 Goethite (**α**-FeOOH)

Pure goethite was synthesized from an alkaline Fe(III) system according the procedure described in Schwertmann and Cornell.[16] Solutions of 1 M $Fe(NO_3)_3$ were prepared by dissolving $Fe(NO_3)_3{\cdot}9H_2O$ in 100 ml UPW in two separate high-density polyethylene (HDPE) bottles with vigorous stirring using a magnetic stir bar. A second solution was prepared by dissolving KOH pellets in UPW to reach 5 M concentration. The KOH solution was rapidly added to the $Fe(NO_3)_3$ solution and immediately filled to 2 L. The HDPE bottles were then closed and stored at 70 °C for 60 hours. Subsequently the supernatant was discarded, and the precipitates washed with UPW to remove OH^- and NO_3^-. After each washing step the suspensions were centrifuged at 4000 rcf for 20 min, the supernatant discarded, and the procedure repeated for a total of 5 rounds. Samples were then dried and stored in a desiccator.

The determined specific surface area was 33.6 m^2 g^{-1}. The XRD pattern aligned well with the reference pattern for goethite in the COD database (Figure S2B).

S2.3 Biogenic magnetite (Fe_3O_4)

Growing the Bacterial Culture

Geobacter sulfurreducens (DSM strain Nr. 12127) was purchased from the German Collection of Microorganisms and Cell Cultures (Leibnitz Institute, DSMZ). To grow the bacteria, air-tight serum bottles were filled with defined growth medium prepared containing acetate (15 mM) as the electron donor and fumarate (40 mM) as the electron acceptor after Coppi et al.[17], closed with bromo-butyl rubber septa and aluminium crimp caps. The pH value was then adjusted to 7 by purging with an 80:20 gas mix of N_2:CO_2 for 45 min and the solutions autoclaved at 121 °C for 30 min. Each serum bottle was inoculated with 10 mL of bacterial culture via syringe and needle. To ensure sterility, the inoculation happened under a Bunsen burner flame and the benchtop, tools and bottle tops were wiped with Ethanol (70 % (v/v)). The bacteria were left to grow overnight at 30 °C in the dark.

A concentrated bacterial stock solution was prepared by combining individual serum bottles. To prevent contact with oxygen, the solutions were purged with N_2 before, during, and after the transfer. The stock solution was then centrifuged at 4900 rcf for 10 min, the supernatant discarded, and the bacteria pellet washed twice with $NaHCO_3$-buffer (30 mM), which had been adjusted to a pH of 7 by purging with N_2 and CO_2 gas for 45 min and autoclaved (121 °C, 30 min). After the last round of centrifuging, the bacteria pellet was mixed with 10 mL of the 30 mM $NaHCO_3$-buffer, transferred into a 20 mL serum bottle, and purged with N_2 gas for 5 min to ensure oxygen-free conditions. The optical density (OD) of this bacteria stock solution was measured with UV-VIS photo spectrometry (Varian Cary 50 Conc).

Magnetite synthesis

The magnetite was synthesized biogenically from 2-line ferrihydrite (Fe(III)-oxyhydroxide) using the previously grown bacterial stocks (*Geobacter sulfurreducens*, DSMZ Nr. 12127). The 2-line ferrihydrite was synthesized with an adjusted protocol from Schwertmann and Cornell[18] by reaction of dissolved $FeCl_3$ with NaOH until pH 7. The precipitated material was washed and centrifuged to remove chloride ions (Cl^-) and characterized using XRD. The synthesis of biogenic magnetite was adjusted from Byrne et al.[19] A solution containing 30 mM $NaHCO_3$ and 20 mM sodium acetate was prepared in air-tight serum bottles closed with bromo-butyl stoppers and aluminium crimp caps, adjusting pH to 7 by purging for 45 min with N_2 and CO_2 gas and subsequent autoclaving (121 °C, 30 mins). The ferrihydrite powder (30 mM) was added under anoxic conditions after autoclaving the solution to prevent structural alterations of the mineral. The bacteria stock solution was added via sterile needle and syringe injection through the stopper to reach an OD of 0.4 in each serum bottle. The suspensions were incubated in the dark at 30 °C for seven days with occasional shaking of the bottles. After incubation the synthesized magnetite was washed five times with $NaHCO_3$ buffer (30 mM, purged with N_2 for 1 h) in an oxygen-free glove box. Instead of centrifuging, a magnet was used for pellet formation. The washed magnetite was then resuspended in 50 ml of the $NaHCO_3$-buffer and stored under anoxic conditions.

The Fe(II)/Fe(III) ratio of the magnetite (BET surface area: 40.8 m^2 g^{-1}) of 0.57 indicated the presence of adsorbed Fe(II), which was extracted from the magnetite surface prior to its use in experiments by adding 0.5 M HCl to the magnetite and letting it sit for 25 minutes.[20] The washed magnetite had a Fe(II)/Fe(III) ratio of 0.45 (stoichiometric magnetite has a ratio of 0.50).

The determined surface area was 40.8 m^2 g^{-1}. The XRD pattern aligned well with the reference pattern for magnetite in the COD database (Figure S2C).

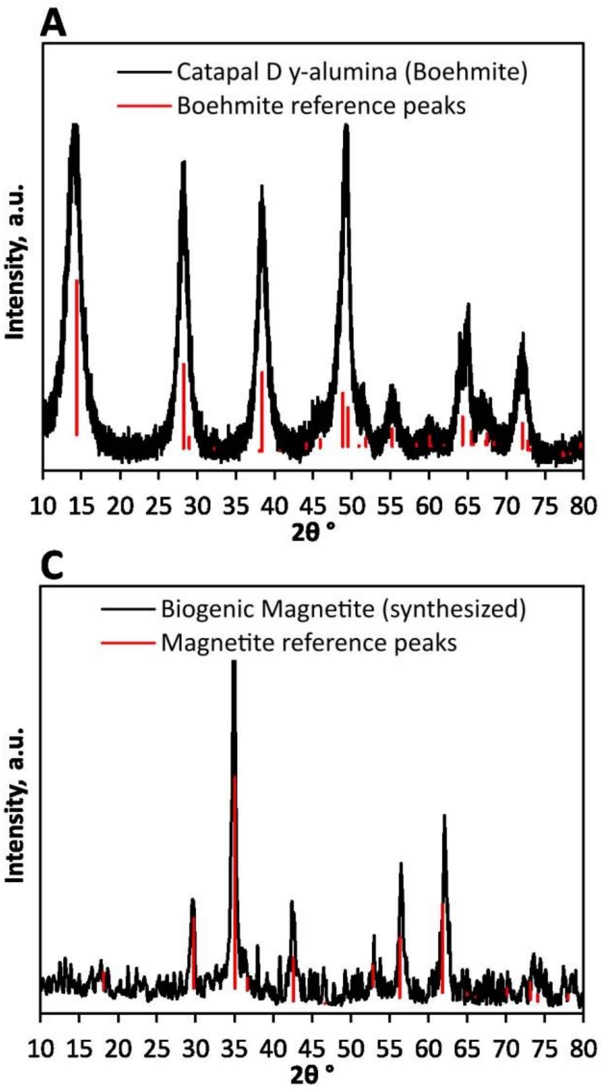

Figure S2. X-ray diffraction patterns for the used minerals **(A)** Catapal D γ-alumina (boehmite) **(B)** goethite **(C)** Biogenically synthesized magnetite

S3 Modeling of Hg and Fe species at initial conditions

Speciation distribution in initial background solutions were calculated using Visual MINTEQ (version 3.1.) using the default thermodynamic database (based on the MINTEQA2 Equilibrium Speciation Model).[21]

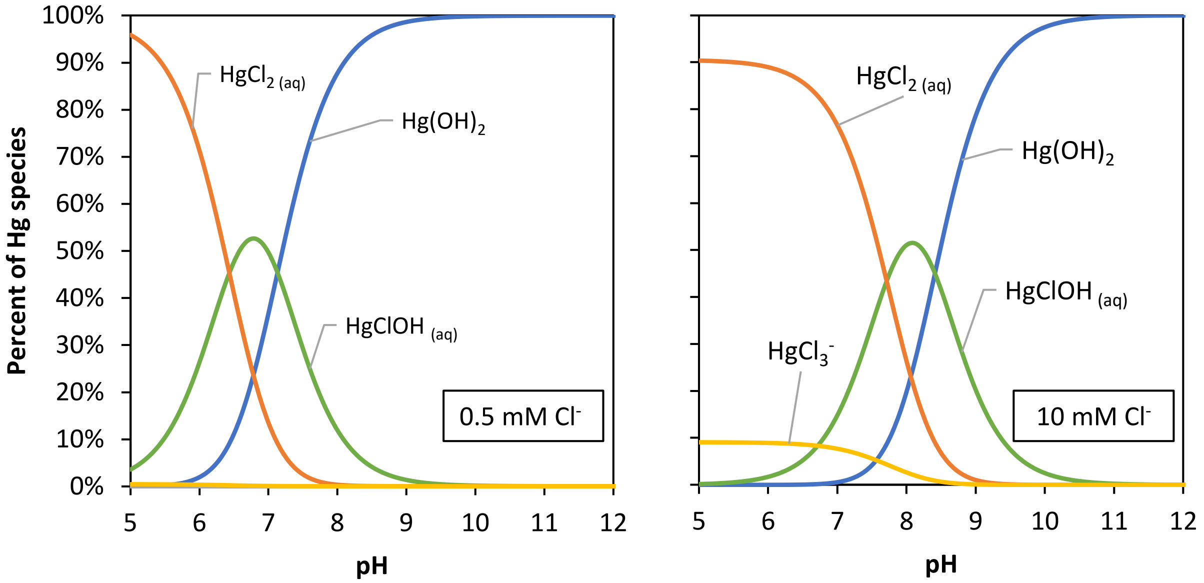

Figure S3.1. Distribution of relevant Hg species at 0.5 mM and 10 mM Cl^- concentration at different pH values. At pH 8 the reactive $Hg(OH)_2$ species makes up 87.8 % at 0.5 mM Cl- and 19.8 % at 10 mM Cl^-. At pH 6.5 the reactive $Hg(OH)_2$ species makes up 11.1 % at 0.5 mM Cl- and 0.06 % at 10 mM Cl^-.

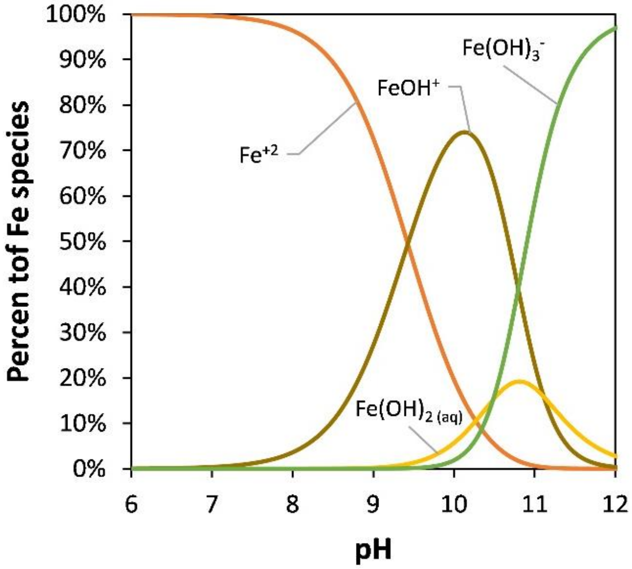

Figure S3.2. Distribution of relevant Fe species at 0.5 mM Cl^- concentration. At pH 8 the reactive $FeOH^+$ species makes up 3.60 % at 0.5 mM Cl^- and 3.07 % at 10 mM Cl^-. At pH 6.5 the reactive $FeOH^+$ species makes up 0.12 % at 0.5 mM Cl^- and 0.10 % at 10 mM Cl^-. This indicates that Fe speciation is less affected by Cl^- compared to Hg(II).

Detailed investigations of the kinetics of Hg(II) reduction by dissolved Fe(II) and fitting of reduction rate expressions showed the best fit when considering only the reactive Fe(II) hydrolysis species $FeOH^+$ and $Hg(OH)_2$.[7] While the Fe^{2+} species can be regarded as nonreactive,[22] for Hg(II) other hydrolysis species (e.g. $HgOH^+$, $Hg(OH)_3^-$) may exhibit different reactivity but the concentration of these species is very low in the pH range (6.5 and 8) used in this study. The increase in Cl^- concentration leads to a shift towards a higher proportion of Hg(II)-chloro complexes, which are less reactive and inhibit Hg(II) reduction.[23] The rate of Hg(II) reduction by dissolved Fe(II) is strongly dependent on pH as can be seen by the relative contribution of the reactive $FeOH^+$ and $Hg(OH)_2$ species to the overall Fe(II) and Hg(II) pool at different pH values (Figure S3.3).

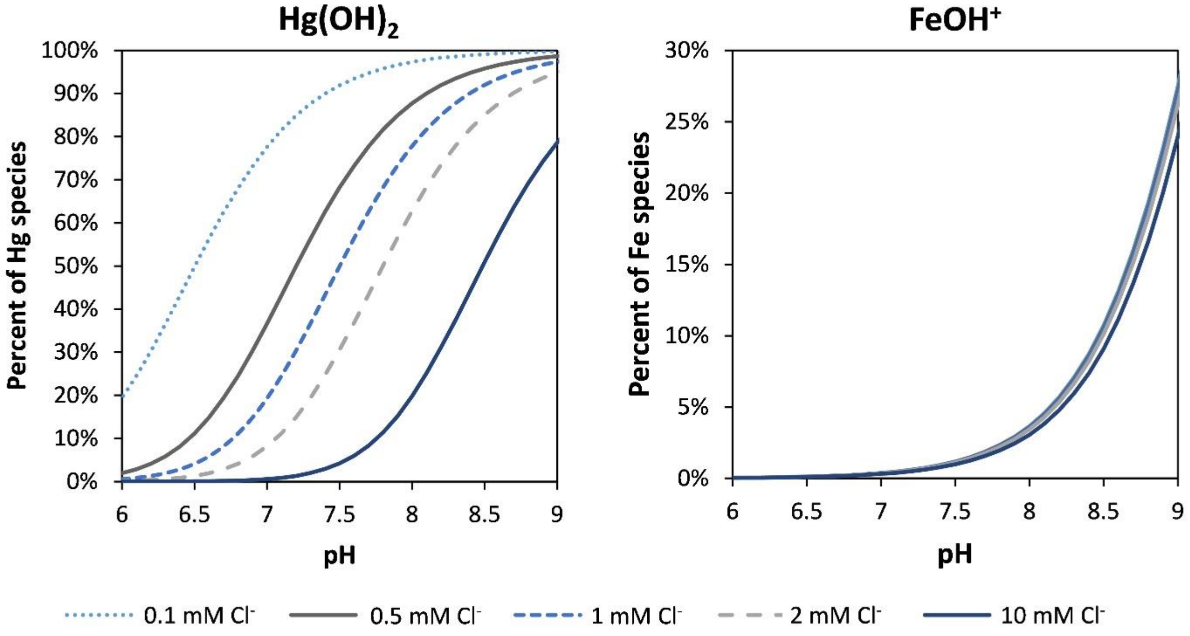

Figure S3.3. Influence of chloride concentrations on the proportion of the reactive $Hg(OH)_2$ and $FeOH^+$ at different pH values.

Table S3. Log equilibrium constants from the MINTEQA2 database used in modeling the species distribution.

Reaction stoichiometry	Log(k)
$Fe^{2+} + H_2O \rightleftharpoons FeOH^+ + H^+$	-9.397
$Fe^{2+} + 2\ H_2O \rightleftharpoons Fe(OH)_2 + 2H^+$	-20.494
$Fe^{2+} + 3\ H_2O \rightleftharpoons Fe(OH)_3^- + 3H^+$	-30.991
$Fe^{2+} + Cl^- \rightleftharpoons FeCl^+$	-0.2
$Hg(OH)_2 + 2\ H^+ \rightleftharpoons Hg^{2+} + 2H_2O$	6.164
$Hg(OH)_2 + H^+ \rightleftharpoons HgOH^+ + 2\ H_2O$	2.767
$Hg(OH)_2 + 2\ H^+ + Cl^- \rightleftharpoons HgCl^+ + 2\ H_2O$	13.49
$Hg(OH)_2 + 2\ H^+ + 2Cl^- \rightleftharpoons HgCl_2 + 2\ H_2O$	20.19
$Hg(OH)_2 + 2\ H^+ + 3Cl^- \rightleftharpoons HgCl_3^- + 2\ H_2O$	21.19
$Hg(OH)_2 + 2\ H^+ + 4\ Cl^- \rightleftharpoons HgCl_4^{2-} + 2\ H_2O$	21.79
$Hg(OH)_2 + H^+ + Cl^- \rightleftharpoons HgClOH + H_2O$	10.444
$2\ Hg(OH)_2 + 3\ H^+ \rightleftharpoons Hg_2OH^{3+} + 3\ H_2O$	9.031

S4 Hg isotope analysis

The concentrations of all samples and standards were matched to 50 nM (10 µg L^{-1}), 25 nM (5 µg L^{-1}), or 10 nM (2 µg L^{-1}) for each analytical session to have signal intensities not differing by more than 10% and an acid strength of < 10 % (v/v). The instrument was tuned daily for optimal signal intensity, stability and peak shapes by adjusting gas flows and electronic settings. Before each analysis a peak centering routine was performed. Data was collected in one block of 36 measurements of 5 s integration time each. The measurement was followed by a washout with 2 % HCl until less than 1 % of the signal intensity was reached. Isotope data are reported as δ-values in permil relative to NIST-3133 calculated according to equation S4.1:

$$\delta^{xxx}Hg\ (‰) = \left(\frac{\frac{^{xxx}Hg}{^{198}Hg} sample}{\frac{^{xxx}Hg}{^{198}Hg} NIST\text{-}3133} - 1 \right) * \ 1000 \qquad (S4.1)$$

Mass independent fractionation (MIF) is reported as deviation from the theoretically predicted mass dependent fractionation (MDF) using the following equation S4.2:

$$\Delta^{xxx}Hg = \ \delta^{xxx}Hg - (\delta^{202}Hg * \beta^{xxx}) \qquad (S4.2)$$

The β^{xxx} values of ^{199}Hg, ^{200}Hg, ^{201}Hg and ^{204}Hg isotopes are 0.2520, 0.5024, 0.7520 and 1.4930, respectively.[24] Assessment of accuracy and precision of the isotope analysis was done by regular measurements of the secondary standard "ETH Fluka". Results were consistent with previous analyses of different laboratories.[25–32] The overall reproducibility (2SD, n = 64) was ± 0.11 ‰ for $\delta^{202}Hg$, ± 0.03 ‰ for $\Delta^{199}Hg$, ± 0.02 ‰ for $\Delta^{200}Hg$, ± 0.02 ‰ $\Delta^{201}Hg$, and ± 0.06 ‰ $\Delta^{201}Hg$ but the standard deviations (2SD) reported for individual sample measurements correspond to the respective session reproducibility of "ETH Fluka" (Table S5).

S5 Assessment of accuracy and precision of isotope analysis

Table S5. Session averages and 2SD values of repeated measurements of the secondary standard "ETH Fluka". The respective session 2SD were used to report uncertainty for samples measured in this session.

Session date	Hg conc. (µg L^{-1})	n	δ^{202}Hg (‰)		Δ^{199}Hg (‰)		Δ^{200}Hg (‰)		Δ^{201}Hg (‰)		Δ^{204}Hg (‰)	
			Average	2SD	Average	2SD	Average	2SD	Average	2SD	Average	2SD
08.12.2021	10	2	-1.46	0.02	0.11	0.02	0.02	0.01	0.03	0.01	-0.02	0.02
15.02.2022	5	6	-1.30	0.04	0.08	0.04	0.02	0.02	0.03	0.03	-0.02	0.06
28.04.2022	10	3	-1.43	0.05	0.07	0.02	0.02	0.02	0.02	0.02	-0.03	0.04
01.06.2022	10	4	-1.33	0.04	0.08	0.02	0.02	0.03	0.02	0.03	0.00	0.06
02.06.2022	10	5	-1.45	0.02	0.09	0.02	0.02	0.02	0.02	0.01	-0.03	0.04
14.06.2022	10	5	-1.43	0.04	0.09	0.02	0.02	0.02	0.03	0.02	-0.03	0.05
06.07.2022	10	7	-1.42	0.09	0.09	0.03	0.02	0.02	0.03	0.03	-0.03	0.04
07.07.2022	5	6	-1.45	0.06	0.08	0.04	0.02	0.04	0.02	0.03	0.01	0.05
26.07.2022	10	6	-1.38	0.06	0.08	0.03	0.02	0.01	0.03	0.02	0.01	0.04
27.07.2022	2.5	5	-1.42	0.08	0.08	0.04	0.02	0.03	0.02	0.02	0.00	0.09
01.09.2022	10	4	-1.44	0.07	0.08	0.03	0.02	0.02	0.03	0.01	-0.01	0.02
04.09.2022	10	4	-1.40	0.02	0.08	0.01	0.02	0.01	0.02	0.03	-0.03	0.06
04.09.2022	5	3	-1.35	0.07	0.09	0.04	0.02	0.04	0.03	0.04	-0.04	0.05
05.09.2022	10	4	-1.45	0.05	0.09	0.04	0.03	0.04	0.02	0.02	-0.04	0.09
**Overall**	**2.5 - 10**	**64**	**-1.41**	**0.11**	**0.08**	**0.03**	**0.02**	**0.02**	**0.03**	**0.02**	**-0.02**	**0.06**

S6 Sorption experiments

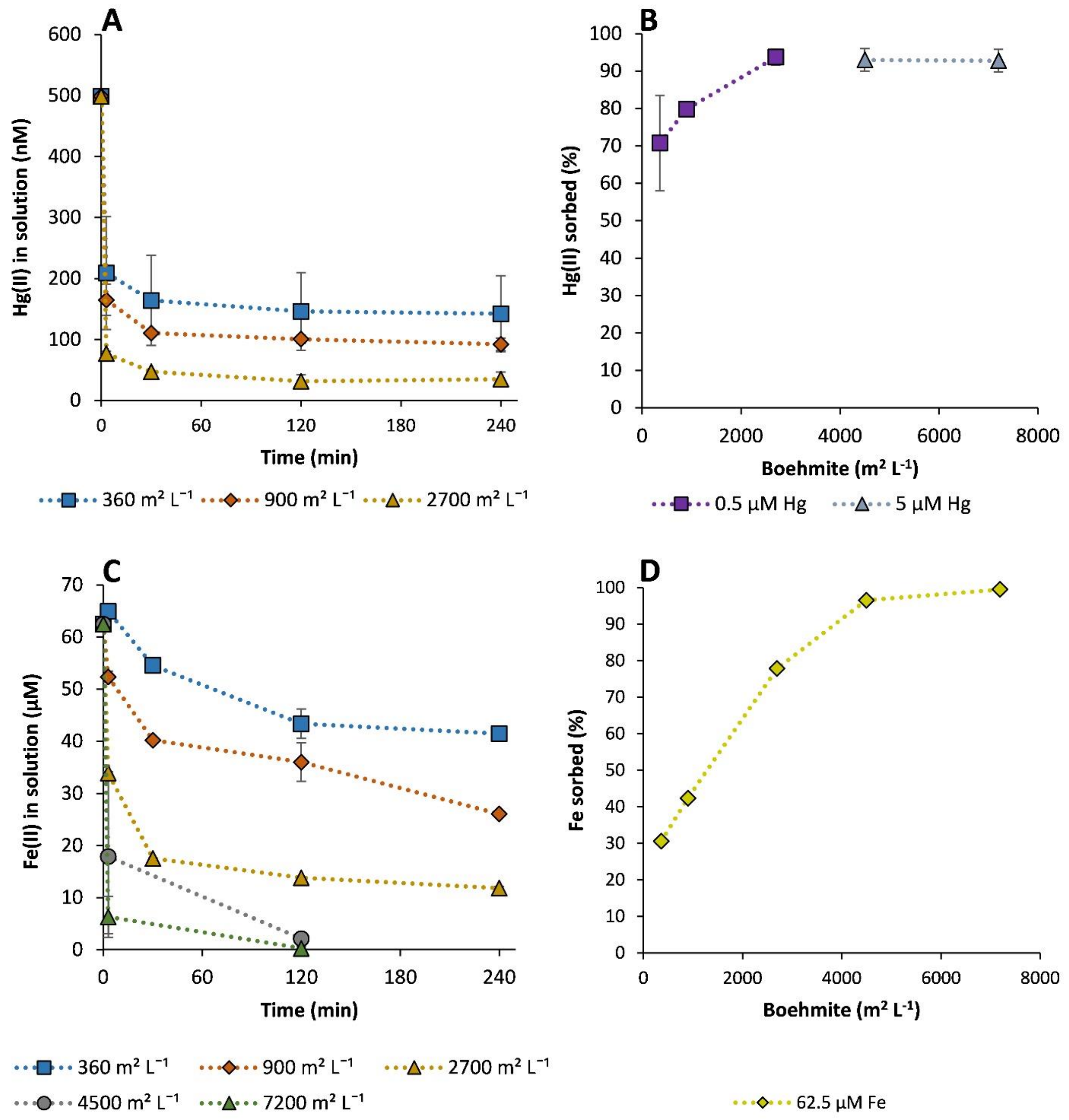

Figure S6. Sorption of Hg(II) to boehmite at 0.5 µM Hg over time **(A)** and after 120 min. of equilibration at different boehmite concentrations **(B)**. Sorption of Fe(II) to boehmite at 62.5 mM Fe over time **(C)** and after 120 min. of equilibration at different boehmite concentrations **(D)**. All experiments were conducted at pH 8. Panels B and D represent the fraction of Hg(II) and Fe(II) sorbed after 120 min of equilibration.

S7 Reduction kinetics and calculation of rate constants

Observed rate constants for second-order reactions of homogenous (k_{hom}) and surface catalyzed experiments (k_{het}) were obtained based on the second order behavior reported in Amirbahman et al.[7]:

$$r_{hom} = k_{hom}[FeOH^{+}][Hg(OH)_2] \quad (S7.1)$$

$$r_{het} = k_{het}[> SOFe_T][Hg(OH)_2] \quad (S7.2)$$

where $>SOFe_T^{(II)}$ is the total sorbed Fe(II). Rate constants for the homogenous reactions were obtained based on thermodynamic calculations of Hg and Fe species distributions in Visual Minteq (Section S3) and by modelling the change of Hg(II) based on the rate law for time increments of 1 minute. A routine was developed using Excel Solver to adjust rate constants by minimizing the sum of squared residuals between the predicted values (Hg(II) remaining; Hg(0) produced) and the experimental data (reactors and traps) at each measured timepoint by predicting the remaining Hg(II) concentration using equation S2:

$$\Delta Hg(II) = k * [FeOH^{+}] * [Hg(OH)_2] * \Delta t \quad (S7.3)$$

The Excel Solver routine was used to fit rate constants for individual experiments as well as for both experimental conditions (0.5 and 10 mM chloride). Fits were calculated using experimental data for reactors (Hg(II) remaining), traps (Hg(0) produced) or reactors and traps together, highlighting the difference in the obtained rate constants depending on the chosen method approach. The Excel Solver function does not provide an estimate about the goodness of the fit. Because of the large pH dependence of Hg(II) reduction by Fe(II) we considered small uncertainties in pH measurements to be the largest source of error in the determination. Measurement of pH in preliminary experiments, without sacrificing the reactors by acidification, resulted in an uncertainty of ± 0.1 pH units. Rate constants were therefore fitted assuming pH 7.9, 8, and 8.1 to estimate the goodness of fit. The reported rate constant represents the mean ± standard deviation of these three fits.

Table S7.1. Rate constants (M^{-1} min^{-1}) for homogeneous Hg(II) reduction experiments by dissolved Fe(II) determined using different method approaches.

Experiment		pH 7.98	pH 8	pH 8.02
Fe(II) 0.5 mM Cl^-	Reactor analysis	-1.65E+04	-1.57E+04	-1.49E+04
	Trap analysis	-1.27E+04	-1.21E+04	-1.15E+04
	Reactor & trap analysis	-1.45E+04	**-1.38E+04**	-1.31E+04
Fe(II) 10 mM Cl^-	Reactor analysis	-3.16E+04	-2.88E+04	-2.62E+04
	Trap analysis	-2.29E+04	-2.08E+04	-1.89E+04
	Reactor & trap analysis	-2.67E+04	**-2.43E+04**	-2.21E+04
Combined	Reactor & trap analysis	-2.03E+04	**-1.88E+04**	-1.75E+04

For the heterogeneous experiments rate constants for kinetic Hg(II) sorption (k_{ads}) and desorption (k_{des}) were included in the Excel Solver routine based on the rate expression reported in Amirbahman et al.[7]

$$r_{ads} = k_{ads}[> SOH][Hg(OH)_2] \quad (S7.4)$$

$$r_{des} = k_{des}[Hg_{ads}] \quad (S7.5)$$

Table S7.2. Fitted rate constants for kinetic Hg(II) adsorption and desorption.

	**Adsorption (k_{ads})**	**Desorption (k_{des})**
**Goethite**	4.76 x 10^{2} M^{-1} min^{-1}	1.30 x 10^{-3} min^{-1}
**Boehmite**	3.80 x 10^{-1} M^{-1} min^{-1}	5.66 x 10^{-4} min^{-1}

Surface H^+ and Fe(II) adsorption were modeled in Visual Minteq using the reaction stoichiometries and log(k) values reported in Amirbahman et al.:[7]

**Reaction stoichiometry**	**Goethite**	**Boehmite**
$>SOH + H^+ \rightleftharpoons >SOH_2^+$	6.19	7.7
$>SOH \rightleftharpoons >SO^- + H^+$	-10.41	-10.2
$>SOH + Fe^{2+} \rightleftharpoons >SOFe^+ + H^+$	-1.29	-3.7
$>SOH + Fe^{2+} + H_2O \rightleftharpoons >SOFe^{(II)}OH + 2H^+$	-9.85	

For pseudo-first-order reactions of Hg(II) with Fe(II)-bearing minerals measured Hg concentrations were linearized by plotting $\ln([Hg^{2+}]_t/[Hg^{2+}]_0)$ as a function of time and obtaining the rate constants from the slope of regressions:[33]

$$k = -\frac{1}{t}\ln\left(\frac{[Hg]_t}{[Hg]_0}\right) \qquad (S7.6)$$

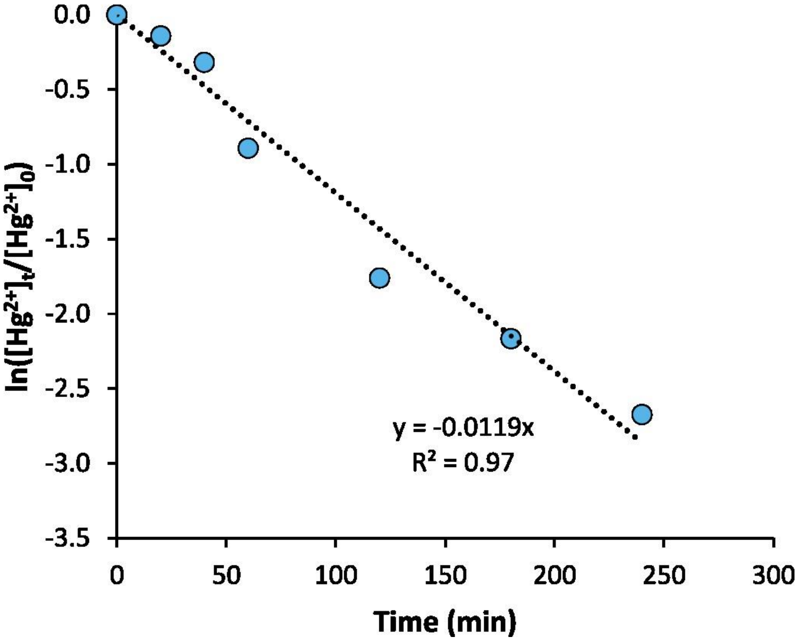

Figure S7. Linearized kinetic data for Hg(II) reduction by magnetite with the dotted line indicating a pseudo-first order law fit.

For comparison a model approach using the Excel Solver function similar to the homogeneous experiments was used to calculate the rate constant by minimizing the sum of squared residuals between predicted Hg concentrations and both reactor and trap measurements:

$$\Delta Hg(II) = k * [Hg] * \Delta t \qquad (S7.7)$$

The resulting rate constant of -1.06 x 10^{-2} min^{-1} was very similar to the one determined using the slope of the linearized concentration data of -1.19 ± 0.06 x 10^{-2} min^{-1}.

Table S7.3. Comparison of reduction kinetics for Hg(II) reduction by Fe(II)-bearing minerals.

	Fe^{II}/Hg^{II} ratio	pH	Chloride (mM)	SSA (m^2 L^{-1})	Fe^{II} surface site (µmol L^{-1})	k_{obs} (x 10^{-3} min^{-1})	k_S (L µmol^{-1} min^{-1})	$t_{1/2}$ (min)
**Magnetite This study**	**74**	**6.5**	**0.5**	**2.0**	**73.8**	**12 ± 0.6**	**0.2**	**58 ± 3**
Magnetite[34]	4’305	6.7	-	2.1	75.2	96	1.3	7
	4’305	6.6	1E-03	2.1	75.2	54	0.7	13
	4’305	6.6	0.1	2.1	75.2	30	0.4	23
	4’305	6.6	10	2.1	75.2	6	0.1	116
	2’152	6.7	-	1.0	37.6	54	1.4	13
	1’076	6.7	-	0.5	18.8	24	1.3	29
	4’305	4.8	-	2.1	75.3	18	0.2	39
	4’305	6.1	-	2.1	75.3	54	0.7	13
Vivianite[35]	1’000	7.0		3.68	36.7	11.7	0.32	59
	100	7.0		0.368	3.67	6.17	1.68	112
	1	7.0		3.68E-03	3.67E-02	1.8	49	385
	0.1	7.0		3.68E-04	3.67E-03	0.14	38	4951
Siderite[36]	31’382	7.5		24.72	246	14.7	0.06	47
	15’691	7.5		12.36	123	20.4	0.17	34
	7’855	7.5		6.18	62	13.9	0.22	50
Green rust[37]	400	8.2		10	83	25	0.30	28
	400	7.0		4.6	38.2	41	1.07	17
	400	8.2		4.6	38.2	16	0.42	43
	400	9.5		4.6	38.2	11	0.29	63

Table S7.4. Remaining Hg(II) and produced Hg(0) fractions during reduction experiments. Measured concentrations were normalized to the initial amount of Hg added to reactors.

Time (h)	Fe(II), kinetic, 0.5 mM chloride			Fe(II), isotope exchange			Fe(II), kinetic, 10 mM chloride			Control (no Fe(II) added)		
	$f_{remaining}$	$F_{reacted}$	mass balance	$f_{reactor}$	f_{trap}	mass balance	$f_{reactor}$	f_{trap}	mass balance	$f_{reactor}$	f_{trap}	mass balance
	105%	0%	105%	106%	0%	106%	98%	0%	98%			
0	99%	0%	99%	106%	0%	106%	100%	0%	100%			
	106%	0%	106%	102%	0%	102%	106%	0%	106%			
	88%	11%	99%	91%	8%	98%	85%	8%	93%			
0.5	85%	8%	93%	83%	5%	89%	85%	9%	94%			
	87%	8%	95%	90%	1%	91%	85%	8%	93%			
	79%	12%	91%	81%	2%	82%	80%	11%	91%			
1	72%	24%	96%	85%	8%	93%	80%	12%	93%			
	77%	12%	89%	80%	9%	89%	77%	14%	91%			
	63%	38%	101%	75%	21%	95%	71%	21%	92%			
2	49%	40%	89%	71%	27%	98%	79%	16%	95%			
	59%	38%	97%	77%	14%	91%	76%	20%	96%			
	31%	70%	101%	46%	31%	77%	57%	33%	91%			
4	29%	66%	94%	50%	30%	81%	55%	36%	91%			
	27%	69%	96%	51%	32%	83%	61%	32%	93%			
	19%	79%	98%	29%	25%	55%	38%	56%	94%			
8	12%	87%	99%	27%	20%	47%	43%	57%	100%			
	8%	83%	92%	34%	28%	62%	37%	60%	97%			
	4%	85%	89%	40%	33%	74%	23%	83%	106%	85%	11%	96%
16	5%	84%	90%	40%	32%	73%	34%	65%	99%	86%	9%	96%
	6%	85%	91%	41%	17%	58%				87%	12%	98%
				42%	22%	64%	21%	79%	100%			
20				46%	31%	77%	19%	71%	90%			
				45%	18%	64%	9%	82%	91%			
							31%	62%	93%			
24							16%	78%	94%			
							13%	78%	91%			

Table S7.4. continued.

Time (h)	Fe(II)-goethite, pH 6.5			Fe(II)-goethite, pH 8			Fe(II)-boehmite, pH 8		
	$f_{remaining}$	$f_{reacted}$	mass balance	$f_{reactor}$	f_{trap}	mass balance	$f_{reactor}$	f_{trap}	mass balance
0	102%		102%	113%	0%	113%	103%	0%	103%
	101%		101%	113%	0%	113%	101%	0%	101%
	99%		99%	111%	0%	111%	103%	0%	103%
0.5	53%	36%	89%	19%	84%	103%	76%	13%	89%
	47%	47%	95%	9%	83%	92%	77%	5%	82%
	47%	42%	89%	7%	88%	95%	82%	12%	94%
1	24%	79%	103%				72%	21%	94%
	21%	79%	100%	8%	83%	91%	75%	21%	97%
	20%	75%	95%	7%	83%	89%	76%	18%	94%
2	16%	84%	100%	9%	84%	92%	61%	29%	90%
	15%	86%	101%				59%	31%	90%
	16%	90%	106%				60%	28%	89%
4	9%	95%	104%	7%	83%	90%	53%	37%	90%
	9%	101%	110%	9%	84%	93%	55%	31%	87%
	10%	100%	110%	9%	84%	93%	58%	33%	90%
8							50%	52%	102%
							50%	49%	99%
							49%	57%	105%
16							41%	40%	81%
							46%	48%	94%
							42%	50%	92%

Table S7.4. continued

Time (h)	$f_{remaining}$	$f_{remaining}$ filtered	Magnetite Hg sorbed	$f_{reacted}$	mass balance
0.08	100%	67%	33%	0%	100%
	100%	42%	58%	0%	100%
	100%	54%	46%	0%	100%
0.33	104%	47%	55%	2%	106%
	70%	28%	59%	10%	80%
	86%	32%	62%	13%	99%
0.67	82%	34%	58%	22%	103%
		17%		36%	
	63%	21%	67%	37%	100%
1	52%	21%	59%	48%	100%
	32%	11%	66%	57%	90%
	38%	12%	68%	64%	102%
2	25%	8%	70%	84%	109%
	13%	4%	69%	84%	97%
	13%	3%	76%	94%	107%
3	18%	3%	82%	98%	117%
	9%	2%	75%	94%	102%
	7%	1%	81%	103%	110%
4	9%	2%	81%	106%	115%
	6%	1%	82%	99%	105%
	6%	1%	88%	107%	113%

S8 Isotope ratios of individual samples

Results of isotope ratio measurements of individual reactor and trap samples. Isotope balances were calculated for timesteps with results for both trap and reactors. The reported 2SD values for isotope balances were propagated from the 2SD values of the reactor and trap measurements as shown in **Equation S8**.

$$2SD\ balance = \sqrt{(2SD\ reactor)^2 + (2SD\ trap)^2} \qquad (S8)$$

Table S8.1. Hg(II) reduction by dissolved Fe(II), isotope exchange (closed system experiment)

Sample	f	δ^{202}Hg (‰)			Δ^{199}Hg (‰)			Δ^{200}Hg (‰)			Δ^{201}Hg (‰)			Δ^{204}Hg (‰)		
		Value	2SD	Balance	Value	2SD	Balance	Value	2SD	Balance	Value	2SD	Balance	Value	2SD [‰]	Balance
R-0-A	1	0.12	0.04		0.01	0.02		0.01	0.02		-0.02	0.02		-0.02	0.05	
R-0.5-A	0.91	0.39	0.04	0.21 (± 0.05)	-0.02	0.02	0.01 (± 0.02)	-0.01	0.02	-0.01 (± 0.02)	-0.02	0.02	-0.01 (± 0.02)	0.02	0.05	0.02 (± 0.07)
T-0.5-A		-1.98	0.04		0.29	0.02		0.04	0.02		0.17	0.02		-0.02	0.05	
R-1-A	0.81	0.37	0.04	0.32 (± 0.07)	-0.06	0.02	-0.05 (± 0.04)	-0.01	0.02	-0.01 (± 0.05)	-0.03	0.02	-0.03 (± 0.03)	0.05	0.05	0.04 (± 0.07)
T-1-A		-2.23	0.06		0.40	0.04		0.09	0.04		0.27	0.03		-0.06	0.05	
R-2-A	0.75	0.64	0.04	0.05 (± 0.05)	-0.09	0.02	-0.01 (± 0.02)	-0.01	0.02	0.00 (± 0.02)	-0.05	0.02	0.00 (± 0.02)	0.00	0.05	0.00 (± 0.07)
T-2-A		-2.09	0.04		0.25	0.02		0.02	0.02		0.16	0.02		-0.04	0.05	
R-4-A	0.46	1.09	0.04	0.07 (± 0.05)	-0.13	0.02	-0.01 (± 0.02)	-0.03	0.02	-0.01 (± 0.02)	-0.10	0.02	-0.01 (± 0.02)	0.04	0.05	0.01 (± 0.07)
T-4-A		-1.45	0.04		0.17	0.02		0.01	0.02		0.12	0.02		-0.04	0.05	
R-8-A	0.40	1.06	0.04	-0.03 (± 0.04)	-0.12	0.02	0.01 (± 0.02)	-0.01	0.02	0.00 (± 0.02)	-0.08	0.02	0.00 (± 0.02)	0.04	0.05	0.01 (± 0.07)
T-8-A		-1.37	0.04		0.16	0.02		0.01	0.02		0.09	0.02		-0.03	0.05	

Table S8.1 continued

Sample	f	δ²⁰²Hg (‰)			Δ¹⁹⁹Hg (‰)			Δ²⁰⁰Hg (‰)			Δ²⁰¹Hg (‰)			Δ²⁰⁴Hg (‰)		
		Value	2SD	Balance	Value	2SD	Balance	Value	2SD	Balance	Value	2SD	Balance	Value	2SD	Balance
R-0-B	1	0.06	0.02		-0.01	0.02		0.00	0.02		-0.01	0.01		0.01	0.04	
R-0.5-B	0.83	0.34	0.02		-0.02	0.02		-0.01	0.02		-0.03	0.01		-0.01	0.04	
T-0.5-B																
R-1-B	0.85	0.34	0.02	0.15	-0.05	0.02	-0.02	-0.01	0.02	-0.01	-0.01	0.01	0.00	0.03	0.04	0.02
T-1-B		-1.92	0.02	(± 0.03)	0.28	0.02	(± 0.03)	0.04	0.02	(± 0.03)	0.16	0.01	(± 0.01)	-0.04	0.04	(± 0.06)
R-2-B	0.71	0.76	0.02	-0.01	-0.08	0.02	0.02	0.00	0.02	0.01	-0.07	0.01	0.00	0.01	0.04	-0.01
T-2-B		-2.06	0.02	(± 0.03)	0.27	0.02	(± 0.03)	0.03	0.02	(± 0.03)	0.16	0.01	(± 0.01)	-0.04	0.04	(± 0.06)
R-4-B	0.50	1.19	0.02	0.13	-0.12	0.02	0.00	-0.01	0.02	0.00	-0.09	0.01	-0.01	0.00	0.04	0.00
T-4-B		-1.63	0.02	(± 0.03)	0.20	0.02	(± 0.03)	0.01	0.02	(± 0.03)	0.12	0.01	(± 0.01)	0.01	0.04	(± 0.06)
R-8-B	0.40	1.31	0.02	0.14	-0.14	0.02	0.01	-0.01	0.02	0.00	-0.09	0.01	0.00	0.00	0.04	-0.01
T-8-B		-1.31	0.02	(± 0.03)	0.20	0.02	(± 0.03)	0.02	0.02	(± 0.03)	0.12	0.01	(± 0.01)	-0.03	0.04	(± 0.06)
R-0-C	1	0.12	0.04		-0.02	0.02		0.01	0.02		0.00	0.02		-0.02	0.05	
R-0.5-C	0.90	0.21	0.04	0.18	-0.03	0.02	-0.02	0.00	0.02	0.00	-0.03	0.02	-0.02	-0.01	0.05	-0.01
T-0.5-C		-2.39	0.06	(± 0.07)	0.38	0.04	(± 0.04)	0.08	0.04	(± 0.05)	0.23	0.03	(± 0.03)	-0.04	0.05	(± 0.07)
R-1-C	0.80	0.49	0.04	0.22	-0.06	0.02	-0.03	0.00	0.02	0.00	-0.04	0.02	-0.02	0.02	0.05	0.01
T-1-C		-2.21	0.04	(± 0.05)	0.30	0.02	(± 0.02)	0.02	0.02	(± 0.02)	0.20	0.02	(± 0.02)	-0.04	0.05	(± 0.07)
R-2-C	0.77	0.66	0.04	0.26	-0.08	0.02	-0.02	-0.01	0.02	-0.01	-0.05	0.02	-0.02	0.00	0.05	-0.01
T-2-C		-1.95	0.04	(± 0.05)	0.29	0.02	(± 0.02)	0.01	0.02	(± 0.02)	0.16	0.02	(± 0.02)	-0.06	0.05	(± 0.07)
R-4-C	0.51	1.15	0.04	0.08	-0.13	0.02	0.01	-0.01	0.02	0.00	-0.08	0.02	0.00	0.02	0.05	-0.01
T-4-C		-1.60	0.04	(± 0.05)	0.22	0.02	(± 0.02)	0.03	0.02	(± 0.02)	0.13	0.02	(± 0.02)	-0.05	0.05	(± 0.07)
R-8-C	0.41	1.04	0.04	0.33	-0.10	0.02	-0.02	-0.01	0.02	0.00	-0.06	0.02	-0.01	-0.01	0.05	-0.02
T-8-C		-1.38	0.04	(± 0.05)	0.17	0.02	(± 0.02)	0.03	0.02	(± 0.02)	0.10	0.02	(± 0.02)	-0.04	0.05	(± 0.07)

Table S8.2 Hg(II) reduction by dissolved Fe(II), 0.5 mM chloride, kinetic (open system experiment)

Sample	f	δ202Hg (‰)			Δ199Hg (‰)			Δ200Hg (‰)			Δ201Hg (‰)			Δ204Hg (‰)		
		Value	2SD	Balance	Value	2SD	Balance	Value	2SD	Balance	Value	2SD	Balance	Value	2SD	Balance
R-0-A	1	0.29	0.06		0.01	0.04		-0.01	0.04		-0.03	0.03		0.01	0.05	
R-0.5-A	0.88	0.56	0.06	0.19	-0.02	0.04	0.01	0.00	0.04	0.00	-0.03	0.03	0.00	0.04	0.05	0.03
T-0.5-A		-2.46	0.06	(± 0.08)	0.25	0.04	(± 0.06)	0.05	0.04	(± 0.06)	0.16	0.03	(± 0.04)	-0.04	0.05	(± 0.07)
R-2-A	0.63	1.36	0.06	0.15	-0.11	0.04	-0.01	-0.02	0.04	-0.02	-0.08	0.03	-0.01	0.03	0.05	-0.02
T-2-A		-1.95	0.06	(± 0.08)	0.17	0.04	(± 0.06)	-0.01	0.04	(± 0.06)	0.10	0.03	(± 0.04)	-0.11	0.05	(± 0.07)
R-4-A	0.31															
T-4-A		-1.49	0.06		0.12	0.04		0.02	0.04		0.09	0.03		0.01	0.05	
R-8-A	0.19	3.59	0.06		-0.28	0.04		-0.02	0.04		-0.22	0.03		-0.02	0.05	
T-8-A																
R-0-B	1	0.01	0.05		0.00	0.02		-0.01	0.02		-0.01	0.02		-0.01	0.04	
R-0.5-B	0.85	0.41	0.05	-0.02	-0.05	0.02	0.00	-0.01	0.02	0.00	-0.04	0.02	-0.01	-0.03	0.04	-0.03
T-0.5-B		-2.46	0.05	(± 0.08)	0.25	0.02	(± 0.03)	0.04	0.02	(± 0.03)	0.16	0.02	(± 0.03)	-0.02	0.04	(± 0.06)
R-2-B	0.49	1.39	0.05	-0.26	-0.12	0.02	0.03	-0.01	0.02	0.02	-0.09	0.02	0.01	0.00	0.04	-0.01
T-2-B		-1.88	0.05	(± 0.08)	0.18	0.02	(± 0.03)	0.04	0.02	(± 0.03)	0.11	0.02	(± 0.03)	-0.02	0.04	(± 0.06)
R-4-B	0.29	3.21	0.05	-0.19	-0.30	0.02	0.03	-0.04	0.02	0.01	-0.20	0.02	0.03	0.05	0.04	0.01
T-4-B		-1.54	0.05	(± 0.08)	0.16	0.02	(± 0.03)	0.03	0.02	(± 0.03)	0.12	0.02	(± 0.03)	-0.01	0.04	(± 0.06)
R-8-B	0.13	4.49	0.05	-0.27	-0.45	0.02	0.04	-0.08	0.02	0.02	-0.26	0.02	0.03	0.06	0.04	-0.01
T-8-B		-0.89	0.05	(± 0.08)	0.11	0.02	(± 0.03)	0.03	0.02	(± 0.03)	0.06	0.02	(± 0.03)	-0.02	0.04	(± 0.06)

Table S8.2. continued

Sample	f	δ202Hg (‰)			Δ199Hg (‰)			Δ200Hg (‰)			Δ201Hg (‰)			Δ204Hg (‰)		
		Value	2SD	Balance	Value	2SD	Balance	Value	2SD	Balance	Value	2SD	Balance	Value	2SD	Balance
R-0-C	1	0.22	0.06		-0.03	0.04		-0.02	0.04		-0.04	0.03		0.02	0.05	
R-0.5-C	0.87	0.55	0.06	0.16 (± 0.08)	-0.09	0.04	-0.04 (± 0.06)	-0.03	0.04	-0.02 (± 0.06)	-0.03	0.03	-0.01 (± 0.04)	0.00	0.05	-0.01 (± 0.07)
T-0.5-C		-2.50	0.06		0.30	0.04		0.04	0.04		0.17	0.03		-0.09	0.05	
R-2-C	0.59	1.39	0.06	0.04 (± 0.08)	-0.13	0.04	0.00 (± 0.06)	-0.02	0.04	-0.01 (± 0.06)	-0.07	0.03	0.00 (± 0.04)	0.01	0.05	0.00 (± 0.07)
T-2-C		-1.92	0.06		0.19	0.04		0.01	0.04		0.10	0.03		-0.03	0.05	
R-4-C	0.27	3.01	0.06	-0.28 (± 0.08)	-0.24	0.04	0.05 (± 0.06)	-0.04	0.04	0.01 (± 0.06)	-0.14	0.03	0.04 (± 0.04)	-0.02	0.05	0.02 (± 0.07)
T-4-C		-1.53	0.06		0.16	0.04		0.03	0.04		0.10	0.03		0.03	0.05	
R-8-C	0.08	4.51	0.06	-0.06 (± 0.08)	-0.41	0.04	0.01 (± 0.06)	-0.06	0.04	0.01 (± 0.06)	-0.29	0.03	0.01 (± 0.04)	0.00	0.05	-0.01 (± 0.07)
T-8-C		-0.48	0.06		0.05	0.04		0.02	0.04		0.04	0.03		-0.01	0.05	

Table S8.3. Hg(II) reduction by dissolved Fe(II), 10 mM chloride, kinetic (open system experiment)

Sample	f	δ^{202}Hg (‰)			Δ^{199}Hg (‰)			Δ^{200}Hg (‰)			Δ^{201}Hg (‰)			Δ^{204}Hg (‰)		
		Value	2SD	Balance	Value	2SD	Balance	Value	2SD	Balance	Value	2SD	Balance	Value	2SD	Balance
R-0-A	1	0.20	0.09		-0.04	0.03		0.00	0.02		-0.01	0.03		-0.02	0.04	
R-0.5-A	0.85	0.43	0.09	0.02 (± 0.12)	-0.04	0.03	0.01 (± 0.04)	0.01	0.02	0.01 (± 0.03)	-0.03	0.03	0.00 (± 0.04)	0.02	0.04	0.01 (± 0.06)
T-0.5-A					0.28	0.03		0.02	0.02		0.16	0.03		-0.07	0.04	
R-1-A	0.80	0.51	0.09	-0.03 (± 0.12)	-0.06	0.03	-0.01 (± 0.04)	0.00	0.02	0.01 (± 0.03)	-0.03	0.03	0.00 (± 0.04)	0.01	0.04	0.00 (± 0.06)
T-1-A					0.23	0.03		0.03	0.02		0.13	0.03		-0.06	0.04	
R-2-A	0.71	0.75	0.09	-0.10 (± 0.12)	-0.09	0.03	0.01 (± 0.04)	0.00	0.02	0.01 (± 0.03)	-0.07	0.03	-0.01 (± 0.04)	0.02	0.04	0.01 (± 0.06)
T-2-A					0.25	0.03		0.05	0.02		0.13	0.03		-0.03	0.04	
R-4-A	0.57	1.26	0.09	-0.14 (± 0.12)	-0.15	0.03	0.00 (± 0.04)	-0.02	0.02	0.01 (± 0.03)	-0.10	0.03	0.01 (± 0.04)	0.02	0.04	-0.01 (± 0.06)
T-4-A					0.21	0.03		0.04	0.02		0.16	0.03		-0.06	0.04	
R-8-A	0.38	2.25	0.09	-0.14 (± 0.12)	-0.26	0.03	0.01 (± 0.04)	-0.04	0.02	0.00 (± 0.03)	-0.15	0.03	0.01 (± 0.04)	0.04	0.04	0.01 (± 0.06)
T-8-A					0.17	0.03		0.03	0.02		0.10	0.03		-0.01	0.04	
R-20-A	0.21	3.37	0.09	0.27 (± 0.09)	-0.36	0.03	-0.04 (± 0.03)	-0.06	0.02	-0.01 (± 0.02)	-0.22	0.03	-0.03 (± 0.03)	0.04	0.04	0.01 (± 0.04)
T-20-A					0.04	0.03		0.00	0.02		0.02	0.03		0.00	0.04	
R-24-A	0.31	2.87	0.05	-0.08 (± 0.10)	-0.32	0.04	0.01 (± 0.05)	-0.03	0.04	0.01 (± 0.05)	-0.20	0.02	0.00 (± 0.03)	0.08	0.09	0.03 (± 0.10)
T-24-A					0.16	0.04		0.03	0.04		0.10	0.02		0.01	0.09	

Table S8.3. continued

Sample	f	$\delta^{202}Hg$ (‰)			$\Delta^{199}Hg$ (‰)			$\Delta^{200}Hg$ (‰)			$\Delta^{201}Hg$ (‰)			$\Delta^{204}Hg$ (‰)		
		Value	2SD	Balance	Value	2SD	Balance	Value	2SD	Balance	Value	2SD	Balance	Value	2SD	Balance
R-0-B	1	0.06	0.04		-0.01	0.02		0.00	0.03		-0.03	0.03		-0.02	0.06	
R-0.5-B	0.85	0.28	0.04	-0.09	-0.03	0.02	0.01	0.00	0.03	0.01	-0.01	0.03	0.01	0.02	0.06	0.02
T-0.5-B		-2.15	0.04	(± 0.06)	0.26	0.02	(± 0.03)	0.04	0.03	(± 0.04)	0.17	0.03	(± 0.05)	0.00	0.06	(± 0.08)
R-1-B	0.80	0.37	0.04	-0.10	-0.05	0.02	0.01	-0.02	0.03	-0.01	-0.03	0.03	0.01	-0.04	0.06	-0.03
T-1-B		-2.08	0.04	(± 0.06)	0.27	0.02	(± 0.03)	0.05	0.03	(± 0.04)	0.17	0.03	(± 0.05)	-0.02	0.06	(± 0.08)
R-2-B	0.79	0.47	0.04	-0.07	-0.06	0.02	0.01	-0.01	0.03	0.00	-0.04	0.03	0.00	0.00	0.06	0.00
T-2-B		-2.05	0.04	(± 0.06)	0.27	0.02	(± 0.03)	0.05	0.03	(± 0.04)	0.16	0.03	(± 0.05)	-0.01	0.06	(± 0.08)
R-4-B	0.55	1.13	0.04	-0.19	-0.13	0.02	0.02	-0.01	0.03	0.00	-0.09	0.03	0.01	0.01	0.06	0.00
T-4-B		-1.82	0.04	(± 0.06)	0.21	0.02	(± 0.03)	0.02	0.03	(± 0.04)	0.13	0.03	(± 0.05)	-0.01	0.06	(± 0.08)
R-8-B	0.43	1.75	0.04	-0.12	-0.21	0.02	0.01	-0.05	0.03	0.00	-0.15	0.03	0.00	0.01	0.06	0.00
T-8-B		-1.54	0.04	(± 0.06)	0.17	0.02	(± 0.03)	0.03	0.03	(± 0.04)	0.11	0.03	(± 0.05)	0.00	0.06	(± 0.08)
R-20-B	0.19	3.46	0.05	-0.16	-0.40	0.04	0.01	-0.06	0.04	0.00	-0.27	0.02	0.02	0.05	0.09	0.01
T-20-B		-0.99	0.04	(± 0.07)	0.10	0.02	(± 0.05)	0.02	0.03	(± 0.05)	0.08	0.03	(± 0.04)	0.00	0.06	(± 0.11)
R-0-C	1	0.27	0.09		0.01	0.03		-0.01	0.02		-0.01	0.03		-0.02	0.04	
R-0.5-C	0.85	0.38	0.09	-0.02	-0.05	0.03	0.00	-0.03	0.02	-0.02	-0.03	0.03	0.00	-0.01	0.04	-0.01
T-0.5-C		-2.33	0.09	(± 0.12)	0.27	0.03	(± 0.04)	0.03	0.02	(± 0.03)	0.17	0.03	(± 0.04)	-0.02	0.04	(± 0.06)
R-1-C	0.77	0.65	0.09	-0.03	-0.06	0.03	0.02	0.00	0.02	0.01	-0.04	0.03	0.01	-0.01	0.04	-0.02
T-1-C		-2.30	0.09	(± 0.12)	0.28	0.03	(± 0.04)	0.06	0.02	(± 0.03)	0.17	0.03	(± 0.04)	-0.05	0.04	(± 0.06)
R-2-C	0.76	0.82	0.09	0.10	-0.11	0.03	-0.02	0.00	0.02	0.01	-0.05	0.03	0.00	0.02	0.04	0.01
T-2-C		-2.15	0.09	(± 0.12)	0.25	0.03	(± 0.04)	0.04	0.02	(± 0.03)	0.15	0.03	(± 0.04)	-0.02	0.04	(± 0.06)
R-4-C	0.61	1.16	0.09	-0.10	-0.15	0.03	0.00	-0.02	0.02	0.00	-0.10	0.03	-0.01	0.01	0.04	-0.02
T-4-C		-2.09	0.09	(± 0.12)	0.25	0.03	(± 0.04)	0.03	0.02	(± 0.03)	0.14	0.03	(± 0.04)	-0.07	0.04	(± 0.06)
R-8-C	0.37	2.49	0.09	-0.03	-0.29	0.03	0.00	-0.03	0.02	0.01	-0.18	0.03	-0.01	0.05	0.04	0.00
T-8-C		-1.50	0.09	(± 0.12)	0.17	0.03	(± 0.04)	0.03	0.02	(± 0.03)	0.08	0.03	(± 0.04)	-0.03	0.04	(± 0.06)
R-20-C	0.09	4.74	0.06	-0.10	-0.48	0.04	0.03	-0.04	0.04	0.02	-0.31	0.03	0.01	0.07	0.05	0.02
T-20-C		-0.58	0.05	(± 0.05)	0.08	0.04	(± 0.04)	0.03	0.04	(± 0.04)	0.04	0.02	(± 0.02)	0.01	0.09	(± 0.09)

Table S8.4. Hg(II) reduction by Fe(II) in presence of boehmite, kinetic (open system experiments)

Sample	f	δ²⁰²Hg (‰)			Δ¹⁹⁹Hg (‰)			Δ²⁰⁰Hg (‰)			Δ²⁰¹Hg (‰)			Δ²⁰⁴Hg (‰)		
		Value	2SD	Balance	Value	2SD	Balance	Value	2SD	Balance	Value	2SD	Balance	Value	2SD	Balance
R-0-A	1	0.10	0.07		0.01	0.03		0.02	0.02		-0.01	0.01		0.00	0.02	
R-0.5-A<br>T-0.5-A	0.76	0.39	0.07	-0.19<br>(± 0.10)	-0.05	0.03	0.01<br>(± 0.04)	0.00	0.02	0.01<br>(± 0.03)	-0.02	0.01	0.02<br>(± 0.02)	0.00	0.02	0.00<br>(± 0.03)
R-1-A<br>T-1-A	0.72	0.62	0.07	-0.07<br>(± 0.10)	-0.08	0.03	0.00<br>(± 0.04)	-0.01	0.02	0.00<br>(± 0.03)	-0.05	0.01	0.00<br>(± 0.02)	-0.02	0.02	-0.02<br>(± 0.03)
R-2-A<br>T-2-A	0.61	0.92	0.07	-0.10<br>(± 0.10)	-0.09	0.03	0.02<br>(± 0.04)	-0.01	0.02	0.01<br>(± 0.03)	-0.05	0.01	0.01<br>(± 0.02)	0.01	0.02	0.00<br>(± 0.03)
R-4-A<br>T-4-A	0.53	1.07	0.07	-0.11<br>(± 0.10)	-0.11	0.03	0.02<br>(± 0.04)	-0.01	0.02	0.00<br>(± 0.03)	-0.06	0.01	0.02<br>(± 0.02)	0.00	0.02	0.02<br>(± 0.03)
R-8-A<br>T-8-A	0.50	1.44	0.07	0.10<br>(± 0.10)	-0.12	0.03	-0.01<br>(± 0.04)	-0.03	0.02	-0.01<br>(± 0.03)	-0.10	0.01	-0.01<br>(± 0.02)	-0.02	0.02	-0.01<br>(± 0.03)
R-16-A<br>T-16-A	0.41	1.54	0.07	-0.28<br>(± 0.10)	-0.16	0.03	0.04<br>(± 0.04)	-0.03	0.02	0.01<br>(± 0.03)	-0.10	0.01	0.03<br>(± 0.02)	-0.03	0.02	0.00<br>(± 0.03)
R-0.5-B<br>T-0.5-B	0.77	0.40	0.05	-0.21<br>(± 0.08)	-0.02	0.04	0.05<br>(± 0.06)	-0.01	0.04	0.01<br>(± 0.05)	0.00	0.02	0.04<br>(± 0.02)	0.00	0.09	0.00<br>(± 0.13)
R-2-B<br>T-2-B	0.59	0.94	0.05	-0.21<br>(± 0.08)	-0.10	0.04	0.03<br>(± 0.06)	-0.03	0.04	0.00<br>(± 0.05)	-0.08	0.02	0.01<br>(± 0.02)	0.01	0.09	0.00<br>(± 0.13)
R-16-B<br>T-16-B	0.46	1.47	0.05	-0.14<br>(± 0.08)	-0.15	0.04	0.01<br>(± 0.06)	-0.01	0.04	0.01<br>(± 0.05)	-0.09	0.02	0.00<br>(± 0.02)	0.03	0.09	0.01<br>(± 0.13)

Table S8.5. Hg(II) reduction by magnetite, kinetic (open system experiments)

Sample	f	δ^{202}Hg (‰)			Δ^{199}Hg (‰)			Δ^{200}Hg (‰)			Δ^{201}Hg (‰)			Δ^{204}Hg (‰)		
		Value	2SD	Balance	Value	2SD	Balance	Value	2SD	Balance	Value	2SD	Balance	Value	2SD	Balance
R-0-C	1	-0.01	0.02		0.00	0.02		-0.01	0.01		0.01	0.01		0.00	0.02	
R-20-C	0.87	0.32	0.02	0.08 (± 0.04)	0.00	0.02	0.02 (± 0.03)	0.01	0.01	0.01 (± 0.02)	-0.01	0.01	0.01 (± 0.02)	0.00	0.02	0.00 (± 0.03)
T-I-20-C		-1.60	0.02		0.19	0.02		0.04	0.01		0.10	0.01		-0.01	0.02	
T-C-20-C*		-1.60	0.02		0.19	0.02		0.04	0.01		0.10	0.01		-0.01	0.02	
R-40-C	0.63	0.91	0.02	0.29 (± 0.06)	-0.06	0.02	-0.01 (± 0.05)	0.00	0.01	0.00 (± 0.03)	-0.04	0.01	-0.01 (± 0.04)	0.05	0.02	0.03 (± 0.07)
T-I-40-C		-1.20	0.04		0.11	0.04		0.00	0.02		0.07	0.03		-0.01	0.06	
T-C-40-C*		-1.34	0.05		0.14	0.05		0.01	0.02		0.08	0.04		-0.01	0.07	
R-60-C	0.38	1.56	0.02	0.40 (± 0.07)	-0.16	0.02	-0.04 (± 0.07)	-0.03	0.01	-0.01 (± 0.03)	-0.10	0.01	-0.03 (± 0.05)	0.07	0.02	0.03 (± 0.10)
T-I-60-C		-0.61	0.04		0.07	0.04		0.02	0.02		0.03	0.03		-0.03	0.06	
T-C-60-C*		-1.04	0.07		0.11	0.06		0.02	0.03		0.06	0.05		-0.02	0.09	
R-120-C	0.13	2.78	0.02	0.29 (± 0.08)	-0.28	0.02	-0.03 (± 0.08)	-0.04	0.01	0.00 (± 0.04)	-0.18	0.01	-0.02 (± 0.06)	0.05	0.02	0.00 (± 0.12)
T-I-120-C		0.26	0.04		-0.02	0.04		0.00	0.02		-0.02	0.03		-0.03	0.06	
T-C-20-C*		-0.67	0.08		0.07	0.07		0.01	0.04		0.04	0.06		-0.02	0.11	
R-180-C	0.07	3.58	0.06	0.35 (± 0.10)	-0.35	0.04	-0.03 (± 0.09)	-0.04	0.04	0.00 (± 0.06)	-0.24	0.03	-0.02 (± 0.07)	0.03	0.05	0.01 (± 0.13)
T-I-180-C		1.31	0.02		-0.18	0.02		-0.05	0.01		-0.10	0.01		0.15	0.02	
T-C-20-C*		-0.55	0.08		0.06	0.08		0.01	0.04		0.03	0.06		-0.01	0.12	
R-240-C	0.50	3.87	0.06	0.36 (± 0.11)	-0.35	0.04	-0.03 (± 0.09)	-0.02	0.04	0.00 (± 0.06)	-0.23	0.03	-0.02 (± 0.07)	0.01	0.05	0.00 (± 0.13)
T-I-240-C		1.88	0.02		-0.30	0.02		-0.03	0.01		-0.22	0.01		0.08	0.02	
T-C-240-C*		-0.51	0.09		0.05	0.08		0.01	0.04		0.02	0.06		-0.01	0.12	

***** In the magnetite experiment the traps were exchanged after each time step. The cumulative product was calculated based on the sum of the traps. The isotope balance accounts for aliquots removed by sampling of reactors.

Table S8.6. Hg(II) reduction by Fe(II) in presence of goethite at pH 6.5, kinetic (open system experiments)

Sample	f	δ^{202}Hg (‰)			Δ^{199}Hg (‰)			Δ^{200}Hg (‰)			Δ^{201}Hg (‰)			Δ^{204}Hg (‰)		
		**Value**	**2SD**	**Balance**	**Value**	**2SD**	**Balance**	**Value**	**2SD**	**Balance**	**Value**	**2SD**	**Balance**	**Value**	**2SD**	**Balance**
R-0-A	1	0.10	0.02		-0.02	0.01		0.01	0.01		-0.03	0.03		-0.02	0.06	
R-0.5-A	0.53	1.19	0.02	-0.14	-0.12	0.01	0.02	0.00	0.01	0.01	-0.08	0.03	0.02	0.02	0.06	0.01
T-0.5-A		-1.67	0.02	(± 0.03)	0.19	0.01	(± 0.02)	0.03	0.01	(± 0.02)	0.13	0.03	(± 0.04)	-0.01	0.06	(± 0.08)
R-1-A	0.24	2.28	0.07	-0.03	-0.29	0.04	-0.01	-0.06	0.04	-0.02	-0.17	0.04	-0.01	0.02	0.05	-0.01
T-1-A		-0.74	0.02	(± 0.08)	0.07	0.01	(± 0.04)	-0.01	0.01	(± 0.04)	0.04	0.03	(± 0.05)	-0.02	0.06	(± 0.08)
R-2-A	0.16	0.96	0.07	-0.03	-0.12	0.04	0.00	0.00	0.04	-0.01	-0.06	0.04	0.00	0.02	0.05	0.01
T-2-A		-0.22	0.02	(± 0.08)	0.03	0.01	(± 0.04)	-0.02	0.01	(± 0.04)	0.01	0.03	(± 0.05)	0.00	0.06	(± 0.08)
R-4-A	0.09	1.40	0.07	-0.04	-0.11	0.04	-0.02	-0.03	0.04	-0.02	-0.11	0.04	0.00	0.03	0.05	0.00
T-4-A		-0.19	0.02	(± 0.08)	-0.01	0.01	(± 0.04)	-0.02	0.01	(± 0.04)	0.01	0.03	(± 0.05)	-0.01	0.06	(± 0.08)
R-0-B	1	0.08	0.02		0.01	0.01		0.01	0.01		-0.03	0.03		-0.01	0.06	
R-0.5-B	0.47	1.39	0.02	-0.16	-0.16	0.01	0.07	-0.03	0.01	0.00	-0.12	0.03	0.03	-0.02	0.06	-0.01
T-0.5-B		-1.55	0.02	(± 0.03)	0.19	0.01	(± 0.02)	0.01	0.01	(± 0.02)	0.10	0.03	(± 0.04)	-0.02	0.06	(± 0.08)
R-1-B	0.21	2.18	0.07	0.00	-0.24	0.04	0.05	-0.04	0.04	0.02	-0.14	0.04	0.03	0.02	0.05	-0.01
T-1-B		-0.59	0.02	(± 0.08)	0.11	0.01	(± 0.04)	0.03	0.01	(± 0.04)	0.06	0.03	(± 0.05)	-0.02	0.06	(± 0.08)
R-2-B	0.15	1.18	0.07	-0.01	-0.14	0.04	0.00	0.00	0.04	-0.02	-0.05	0.04	0.00	-0.01	0.05	-0.01
T-2-B		-0.22	0.02	(± 0.08)	0.02	0.01	(± 0.04)	-0.02	0.01	(± 0.04)	0.01	0.03	(± 0.05)	-0.01	0.06	(± 0.08)
R-4-B	0.19	1.31	0.07	-0.06	-0.16	0.04	0.03	-0.02	0.04	0.01	-0.10	0.04	0.02	0.04	0.05	0.02
T-4-B		-0.20	0.02	(± 0.08)	0.03	0.01	(± 0.04)	0.01	0.01	(± 0.04)	0.02	0.03	(± 0.05)	0.02	0.06	(± 0.08)

Table S8.6. continued

Sample	f	$\delta^{202}Hg$ (‰)			$\Delta^{199}Hg$ (‰)			$\Delta^{200}Hg$ (‰)			$\Delta^{201}Hg$ (‰)			$\Delta^{204}Hg$ (‰)		
		**Value**	**2SD**	**Balance**	**Value**	**2SD**	**Balance**	**Value**	**2SD**	**Balance**	**Value**	**2SD**	**Balance**	**Value**	**2SD**	**Balance**
R-0-C	1	0.03	0.05		-0.01	0.04		-0.01	0.04		-0.01	0.02		-0.01	0.09	
R-0.5-C	0.47	1.56	0.05	-0.02	-0.17	0.04	0.02	-0.03	0.04	0.00	-0.12	0.02	0.00	0.04	0.09	0.00
T-0.5-C		-1.40	0.05	(± 0.08)	0.18	0.04	(± 0.06)	0.03	0.04	(± 0.05)	0.12	0.02	(± 0.02)	-0.02	0.09	(± 0.13)
R-1-C	0.20	2.18	0.07	-0.08	-0.23	0.04	0.01	-0.02	0.04	0.01	-0.17	0.04	0.00	0.03	0.05	0.04
T-1-C		-0.64	0.05	(± 0.09)	0.07	0.04	(± 0.05)	0.02	0.04	(± 0.06)	0.05	0.02	(± 0.04)	0.04	0.09	(± 0.11)
R-2-C	0.16	1.30	0.07	0.07	-0.11	0.04	0.02	-0.02	0.04	0.01	-0.11	0.04	0.00	-0.03	0.05	0.00
T-2-C		-0.17	0.05	(± 0.09)	0.05	0.04	(± 0.05)	0.02	0.04	(± 0.06)	0.02	0.02	(± 0.04)	0.01	0.09	(± 0.11)
R-4-C	0.10	1.37	0.07	0.01	-0.12	0.04	0.01	-0.01	0.04	-0.01	-0.10	0.04	0.00	0.01	0.05	0.01
T-4-C		-0.14	0.05	(± 0.09)	0.02	0.04	(± 0.05)	-0.01	0.04	(± 0.06)	0.01	0.02	(± 0.04)	0.01	0.09	(± 0.11)

Table S8.7. Hg(II) reduction by Fe(II) in presence of goethite at pH 8, kinetic (open system experiments)

Sample	f	δ^{202}Hg (‰)			Δ^{199}Hg (‰)			Δ^{200}Hg (‰)			Δ^{201}Hg (‰)			Δ^{204}Hg (‰)		
		Value	2SD	Balance	Value	2SD	Balance	Value	2SD	Balance	Value	2SD	Balance	Value	2SD	Balance
R-0-A	1	0.05	0.06		-0.02	0.03		0.00	0.01		0.00	0.02		0.00	0.04	
R-0.5-A	0.19	1.52	0.06	0.19 (± 0.09)	-0.17	0.03	-0.02 (± 0.04)	-0.03	0.01	0.00 (± 0.02)	-0.10	0.02	-0.02 (± 0.02)	-0.01	0.04	0.01 (± 0.06)
T-0.5-A		-0.12	0.06		0.01	0.03		0.00	0.01		0.00	0.02		0.01	0.04	
R-2-A	0.09	0.65	0.08	-0.02 (± 0.10)	-0.09	0.04	-0.01 (± 0.05)	0.03	0.03	0.00 (± 0.03)	-0.03	0.02	0.00 (± 0.03)	0.04	0.09	0.02 (± 0.10)
T-2-A		-0.08	0.06		0.00	0.03		0.00	0.01		0.01	0.02		0.02	0.04	
R-4-A	0.07	0.70	0.08	-0.05 (± 0.10)	-0.07	0.04	0.03 (± 0.05)	0.01	0.03	0.01 (± 0.03)	-0.01	0.02	0.01 (± 0.03)	0.07	0.09	0.02 (± 0.10)
T-4-A		-0.11	0.06		0.03	0.03		0.01	0.01		0.01	0.02		0.02	0.04	
R-0-B	1	0.05	0.06		-0.02	0.03		-0.02	0.01		-0.01	0.02		-0.02	0.04	
R-0.5-B	0.09	-0.08	0.06	-0.15 (± 0.09)	-0.02	0.03	0.02 (± 0.04)	0.00	0.01	-0.01 (± 0.02)	-0.03	0.02	0.01 (± 0.02)	-0.04	0.04	-0.01 (± 0.06)
T-0.5-B		-0.15	0.06		0.02	0.03		-0.01	0.01		0.01	0.02		-0.01	0.04	
R-1-B	0.08	0.88	0.08	-0.17 (± 0.10)	-0.12	0.04	0.02 (± 0.05)	-0.02	0.03	-0.01 (± 0.03)	-0.13	0.02	0.02 (± 0.03)	-0.05	0.09	-0.01 (± 0.10)
T-1-B		-0.27	0.06		0.03	0.03		-0.01	0.01		0.03	0.02		-0.01	0.04	
R-4-B	0.09															
T-4-B		-0.10	0.06		0.03	0.03		0.01	0.01		0.02	0.02		0.00	0.04	
R-0-C	1	0.06	0.06		0.02	0.03		0.01	0.01		0.02	0.02		-0.04	0.04	
R-0.5-C	0.07	0.03	0.06	-0.18 (± 0.09)	-0.03	0.03	0.02 (± 0.04)	-0.01	0.01	-0.01 (± 0.02)	-0.05	0.02	0.01 (± 0.02)	-0.03	0.04	-0.02 (± 0.06)
T-0.5-C		-0.20	0.06		0.03	0.03		-0.01	0.01		0.01	0.02		-0.01	0.04	
R-1-C	0.07															
T-1-C		-0.19	0.06		0.02	0.03		-0.01	0.01		0.03	0.02		0.02	0.04	

S9 Odd and even mass MIF

The two plausible mechanisms explaining MIF in the Hg isotope system are the nuclear volume effect (NVE) and the magnetic isotope effect (MIE). Only a small part of the NVE is mass-independent and apart from small anomalies, the nuclear charge radius, and thus the nuclear volume, expands nearly linearly with isotopic mass.[38] In equilibrium reactions the fractionation caused by the NVE can be as large as by the mass difference effect (MDE) and both, NVE and MDE, enrich light isotopes in the produced Hg(0).[39,40] The odd-number isotopes have slightly smaller nuclear charge radii than predicted by a linear relationship with mass (based on ^{198}Hg and ^{202}Hg) and this deviation from mass-dependent scaling leads to the mass-independent component of the NVE effect (Section S9).[41] Additionally, small deviations from this linear relationship have been theoretically predicted for the even-mass isotopes ^{200}Hg and ^{204}Hg (Section S9).[42] The MIE is completely independent of isotopic mass and only occurs in kinetically controlled reactions under specific conditions such as during photochemical reactions.[38,41] The MIE only affects the odd-mass Hg isotopes, which have nonzero nuclear spin and nuclear magnetic moments. In MIE the magnetic and nonmagnetic isotopes can preferentially accumulate in different reaction products during reactions involving excited radical-pair intermediates that undergo spin conversion, such as photoreduction.[43,44] Photoreduction can result in both enrichment and depletion of odd-mass Hg isotopes.[45] In addition to NVE and MIE alternative potential mechanisms for even-mass MIF observed in rainfall and snow[46–49] have been proposed recently.[50,51]

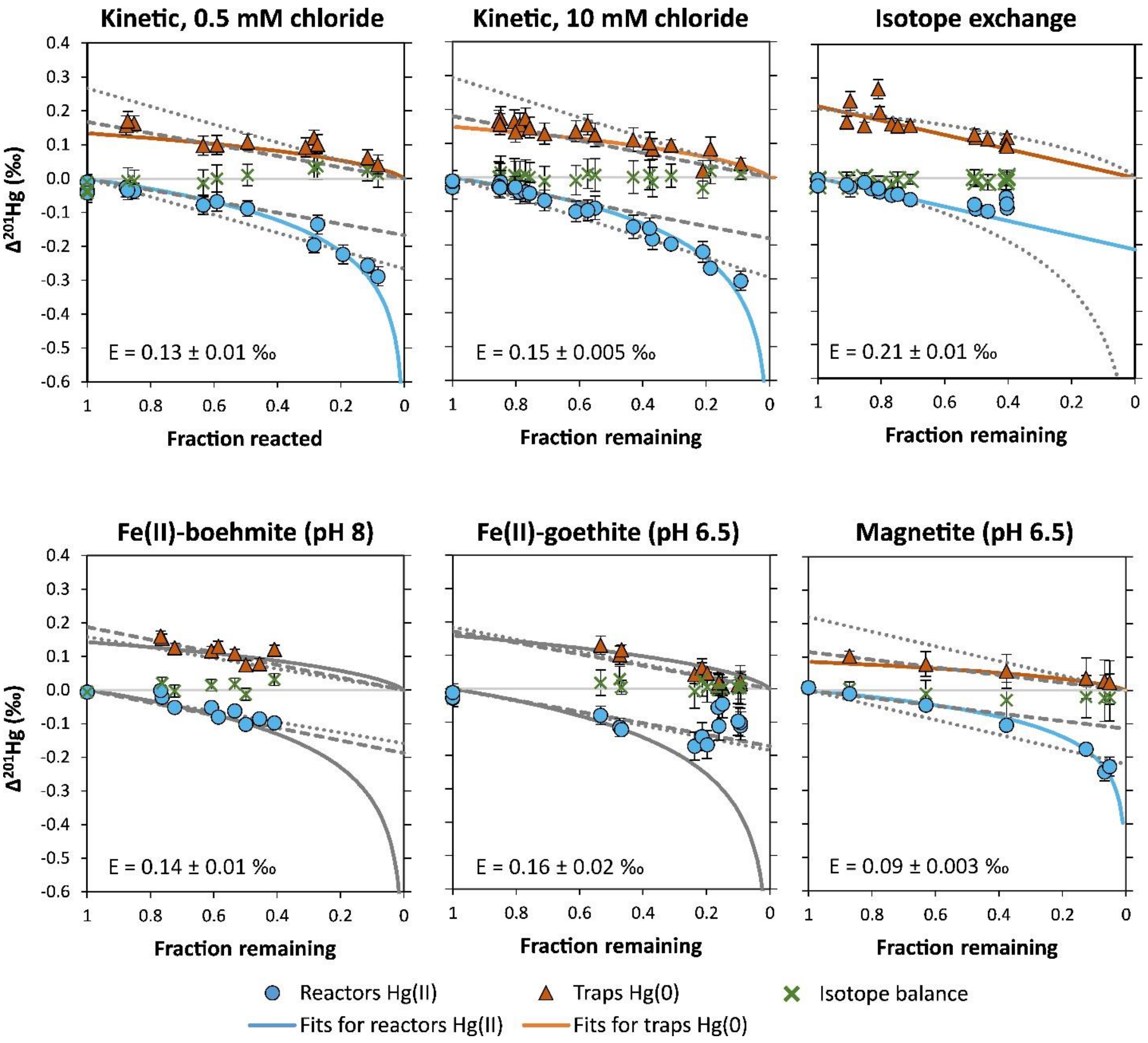

Figure S9.1. Odd mass MIF ($\Delta^{201}Hg$) during Hg(II) reduction experiments. For kinetic experiments a Rayleigh model and for isotope exchange experiments an equilibrium model was fitted. Rayleigh model fits are represented by solid lines, linear equilibrium model fits based on reactors are represented by dotted and fits based on traps by dashed lines, respectively. For the isotope exchange experiment, the dotted lines show a Rayleigh model fit.

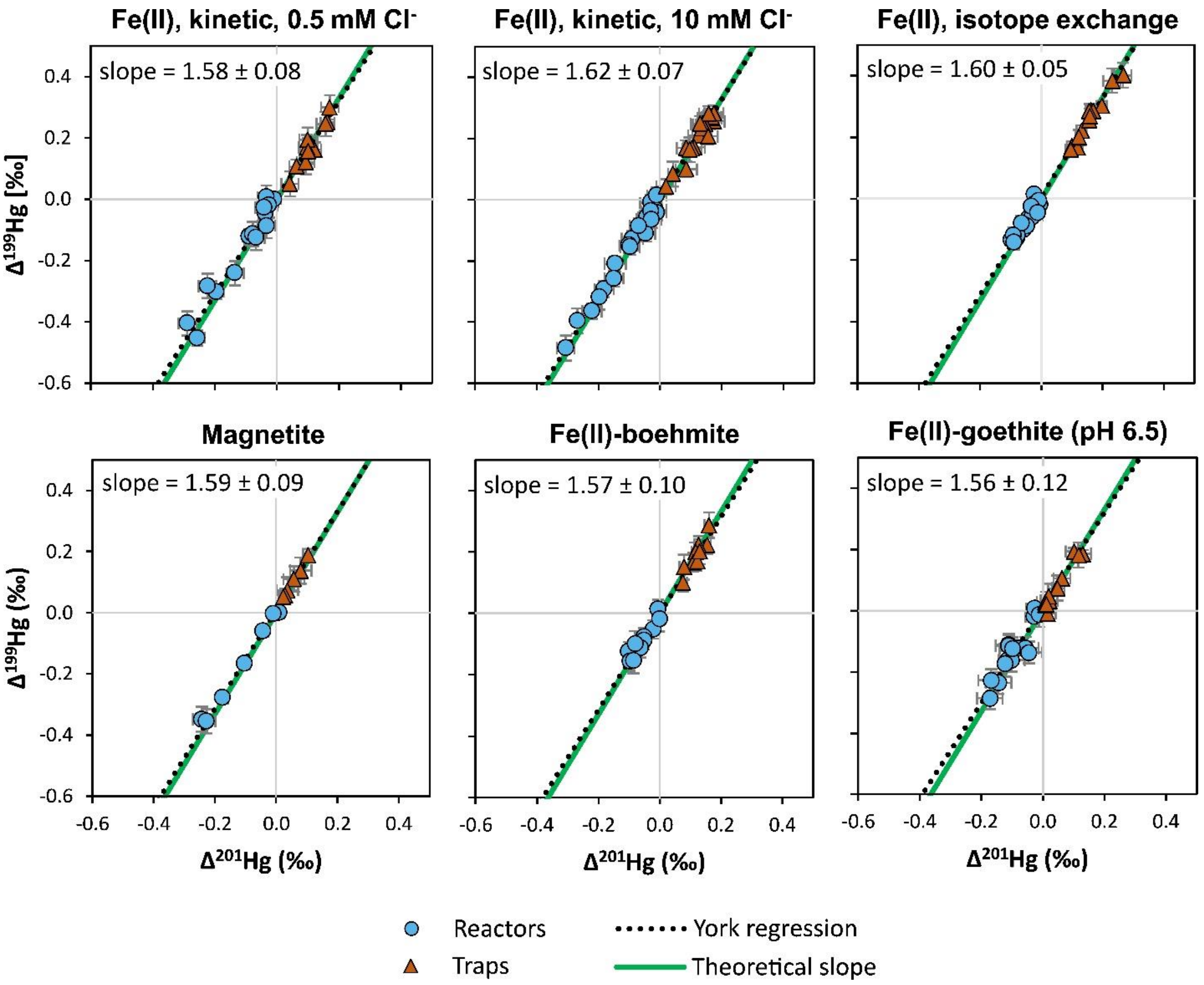

Figure S9.2. Relationship between Δ^{201}Hg and Δ^{199}Hg for Hg(II) reduction experiments. The dotted lines represent York regressions calculated in R using the IsoplotR package.[52] Error bars represent 2SD and are mostly smaller than the data symbol. The theoretically predicted slope for the NVE is 1.65.[40]

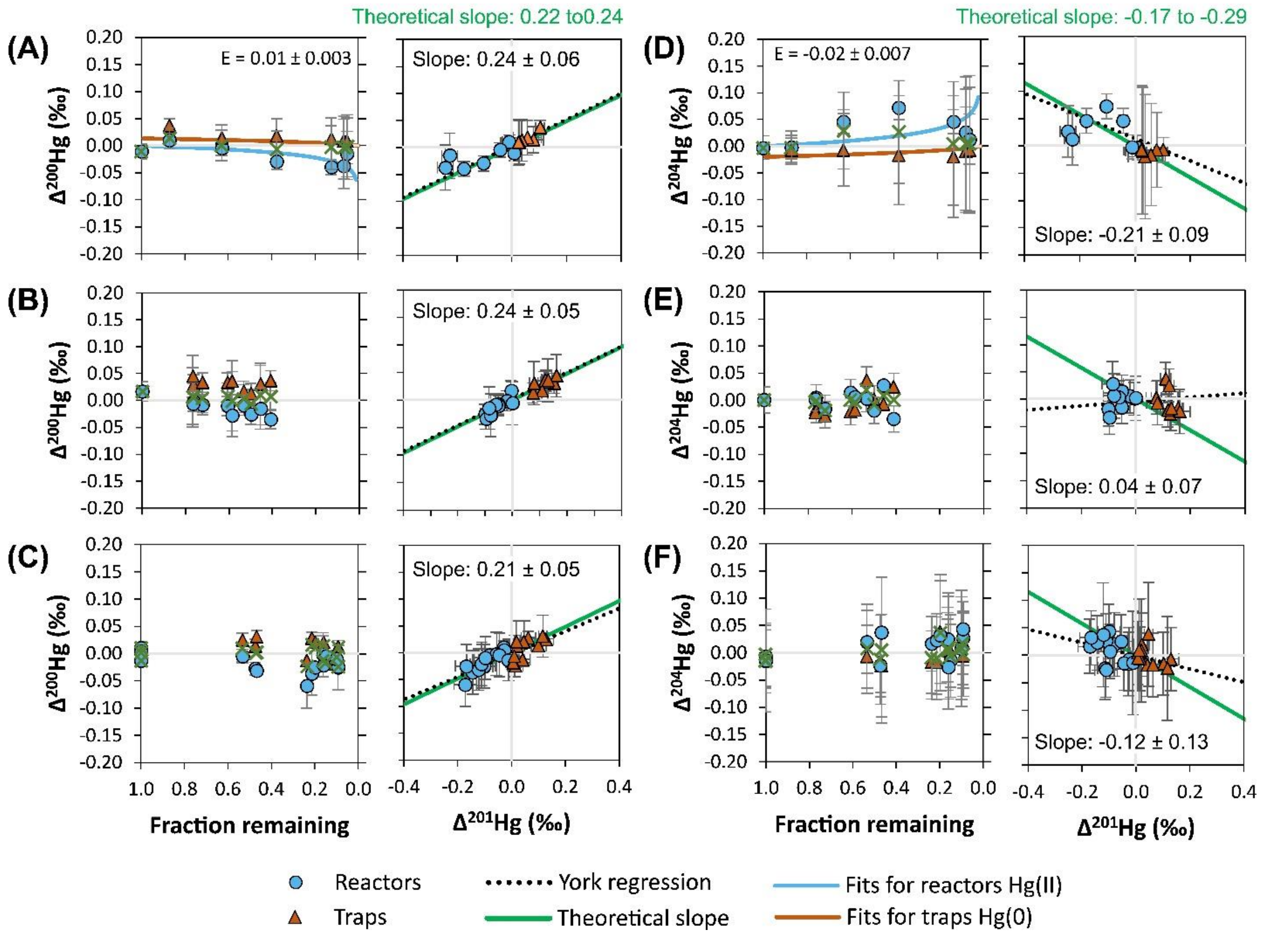

Figure S9.3. Mass-independent fractionation of ^{200}Hg for **(A)** magnetite experiments **(B)** Fe(II)-boehmite experiments and **(C)** Fe(II)-goethite experiments. Mass-independent fractionation of ^{204}Hg in **(D)** magnetite experiments **(E)** Fe(II)-boehmite experiments **(F)** Fe(II)-goethite experiments. The dotted lines represent York regressions calculated in R using the IsoplotR package.[52]

Table S9.1. Comparison of nuclear charge radii for stable Hg isotopes.

Hg isotope	Exact mass	R [fm] Fricke & Heilig[53]	R [fm] Angeli-2013[54]	R [fm] Nadjakov[55]
196	195.9658	5.435	5.4385	5.4380
198	197.9668	5.443	5.4463	5.4455
199	198.9683	5.444	5.4474	5.4467
200	199.9683	5.452	5.4551	5.4542
201	200.9703	5.455	5.4581	5.4571
202	201.9706	5.462	5.4648	5.4637
204	203.9735	5.472	5.4744	5.4730

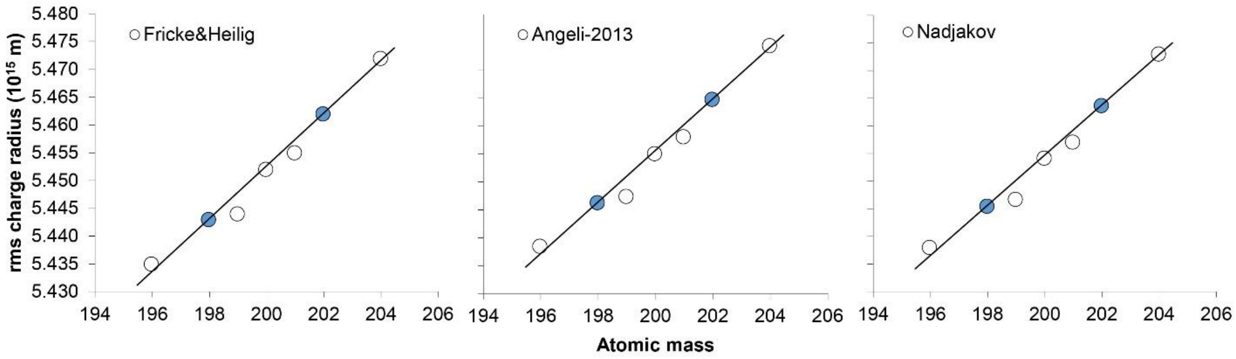

Figure S9.4. Comparison of root mean squared (rms) nuclear charge radii from three literature sources. Lines show linear trends based on $^{202/198}$Hg.

Table S9.2. Comparison of scaling factors for Hg isotope ratios relative to $^{202/198}$Hg

	$^{196/198}$Hg	$^{199/198}$Hg	$^{200/198}$Hg	$^{201/198}$Hg	$^{202/198}$Hg	$^{204/198}$Hg
MDF-kinetic	-0.5074	0.2520	0.5024	0.7520	1.0000	1.4928
MDF-equilibrium	-0.5151	0.2539	0.5049	0.7539	1.0000	1.4855
NVF (Fricke & Heilig)[53]	-0.4200	0.0525	0.4732	0.6312	1.0000	1.5277
NVF (Angeli-2013)[54]	-0.4206	0.0594	0.4753	0.6374	1.0000	1.5203
NVF (Nadjakov)[55]	-0.4111	0.0658	0.4776	0.6370	1.0000	1.5123

Table S9.3. Theoretical slopes for the ratios ("slopes") of Δ^{199}Hg, Δ^{200}Hg, and Δ^{204}Hg versus Δ^{201}Hg, as well as Δ^{200}Hg versus Δ^{204}Hg.

	$^{199/201}$Hg "slope"	$^{200/201}$Hg "slope"	$^{204/201}$Hg "slope"	$^{200/204}$Hg "slope"
Fricke & Heilig[53]	1.6506	0.2413	-0.2888	-0.8355
Angeli-2013[54]	1.6814	0.2370	-0.2395	-0.9896
Nadjakov[55]	1.6183	0.2156	-0.1692	-1.2742

Table S9.4. Calculated contribution of NVE to total Hg isotope effect for $^{202/198}Hg$ (data from Wiederhold et al.[40])

	Sum [‰][40]	NVE [‰][40]	MDE [‰][40]	NVE contribution
**$Hg(SMe)^+$**	1.04	0.79	0.26	76 %
**$Hg(SH)^+$**	1.28	0.97	0.31	76 %
**$Hg(SMe)_2$**	1.53	0.82	0.70	54 %
**$Hg(SH)_2$**	1.63	0.91	0.73	55 %
**HgSMeCl**	1.78	0.98	0.80	55 %
**HgSHCl**	1.80	1.04	0.76	58 %
**$HgOH^+$**	1.82	1.41	0.42	77 %
**HgSHOH**	1.88	0.96	0.92	51 %
**$HgCl^+$**	1.88	0.96	0.92	51 %
**HgSMeOH**	1.90	0.90	1.00	47 %
**$HgCl_2$**	2.09	1.25	1.11	60 %
**HgClOH**	2.17	1.11	1.06	51 %
**$Hg(OH)_2$**	2.19	1.00	1.19	46 %
**$HgCl_4^{2-}$**	2.63	2.23	0.40	85 %

Theoretical nuclear volume effect (NVE) driven MIF enrichment factors were calculated based on reported nuclear charge radii (Table S9.1), scaling factors (SF) for Hg isotopes relative to $^{202/198}Hg$ (Table S9.2) and the computational data for 1000 ln β values (≈ ε-values) for different Hg species relative to Hg(0) vapor[40] (Table S9.4) according to equation S6:

$$E^{XXX}Hg = (\varepsilon^{202}Hg_{NVF} * SF_{NVF}^{XXX}) - (\varepsilon^{202}Hg_{NVF} * SF_{MDF}^{XXX}) \qquad (S6)$$

Table S9.5. Calculated NVE driven MIF enrichment factors for equilibrium fractionation relative to Hg(0) vapor ($E^{XXX}Hg$) based on nuclear charge radii from Fricke & Heilig (F&H)[53], Angeli-2013 (A)[54] and Nadjakov (N).[55] The value shown in bulk font were used for the calculations in this study.

	$E^{199}Hg$ [‰]			$E^{200}Hg$ [‰]			$E^{201}Hg$ [‰]			$E^{204}Hg$ [‰]		
	F&H[53]	A[54]	N[55]	F&H[53]	A[54]	N[55]	F&H[53]	A[54]	N[55]	F&H[53]	A[54]	N[55]
**$Hg(SMe)^+$**	-0.157	-0.151	-0.146	-0.023	-0.021	-0.019	-0.095	-0.090	-0.090	0.027	0.022	0.015
**$Hg(SH)^+$**	-0.194	-0.187	-0.181	-0.028	-0.026	-0.024	-0.118	-0.111	-0.112	0.034	0.027	0.019
**$Hg(SMe)_2$**	-0.165	-0.159	-0.154	-0.024	-0.022	-0.020	-0.100	-0.095	-0.095	0.029	0.023	0.016
**$Hg(SH)_2$**	-0.181	-0.174	-0.169	-0.026	-0.025	-0.022	-0.109	-0.104	-0.104	0.032	0.025	0.018
**HgSMeCl**	-0.195	-0.188	-0.182	-0.028	-0.027	-0.024	-0.118	-0.112	-0.112	0.034	0.027	0.019
**HgSHCl**	-0.207	-0.200	-0.193	-0.030	-0.028	-0.026	-0.126	-0.119	-0.120	0.036	0.029	0.020
**$HgOH^+$**	-0.281	-0.271	-0.262	-0.041	-0.038	-0.035	-0.170	-0.161	-0.162	0.049	0.039	0.027
**HgSHOH**	-0.191	-0.184	-0.178	-0.028	-0.026	-0.024	-0.115	-0.109	-0.110	0.033	0.026	0.019
**$HgCl^+$**	-0.297	-0.287	-0.278	-0.043	-0.040	-0.037	-0.180	-0.171	-0.172	0.052	0.041	0.029
**HgSMeOH**	-0.180	-0.173	-0.168	-0.026	-0.024	-0.022	-0.109	-0.103	-0.104	0.031	0.025	0.018
**$HgCl_2$**	**-0.249**	-0.241	-0.233	**-0.036**	-0.034	-0.031	**-0.151**	-0.143	-0.144	**0.044**	0.034	0.024
**HgClOH**	**-0.222**	-0.214	-0.207	**-0.032**	-0.030	-0.028	**-0.134**	-0.127	-0.128	**0.039**	0.031	0.022
**$Hg(OH)_2$**	**-0.199**	-0.192	-0.186	**-0.029**	-0.027	-0.025	**-0.121**	-0.114	-0.115	**0.035**	0.027	0.019
**$HgCl_4^{2-}$**	-0.445	-0.430	-0.415	-0.065	-0.061	-0.055	-0.270	-0.256	-0.257	0.078	0.061	0.043

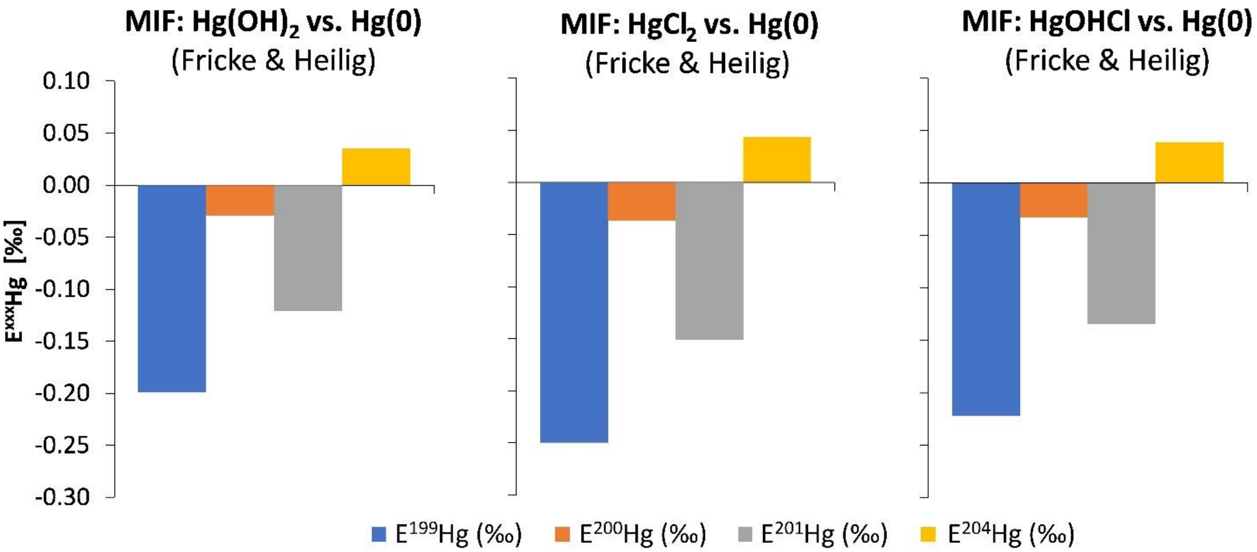

Figure S9.5. Comparison of the extent and direction of odd- and even-mass MIF predicted by the nuclear volume effect for the dominant Hg(II) species under the experimental conditions of this study relative to Hg(0) vapor. The predicted extent of MIF is based on the nuclear charge radii reported in Fricke & Heilig.[53]

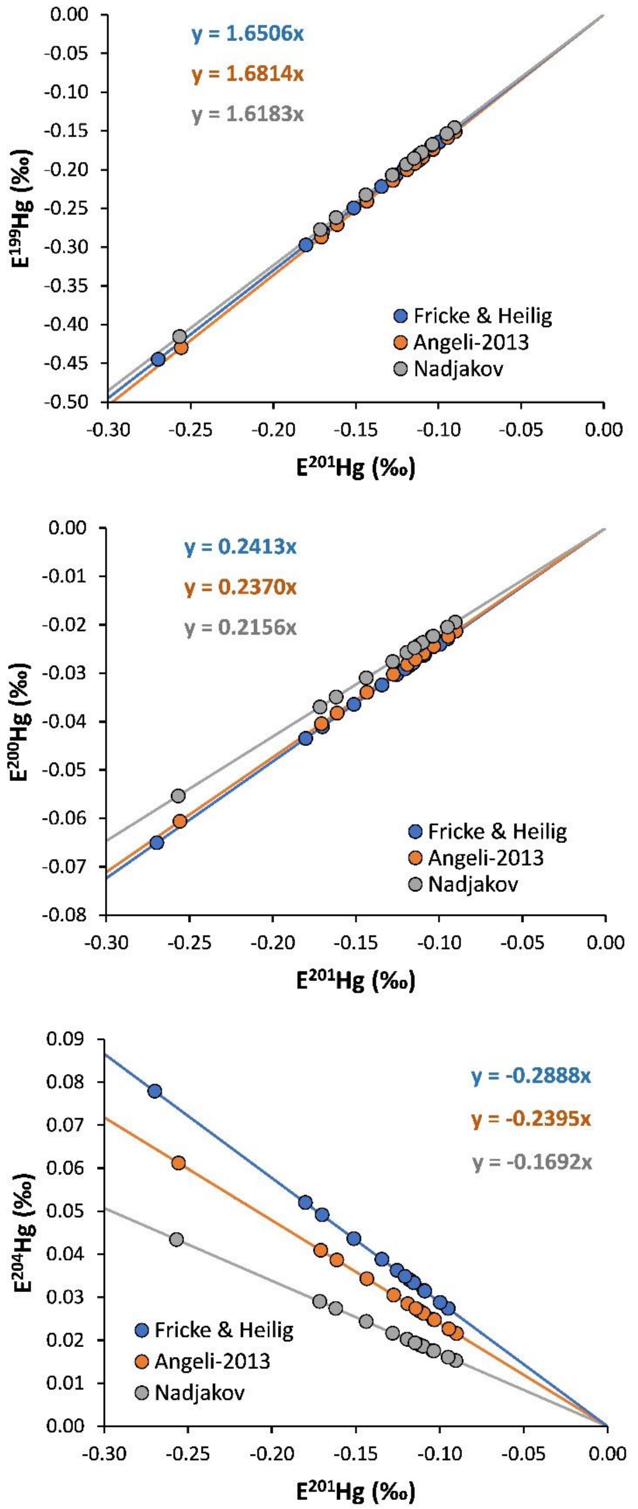

Figure S9.6. Comparison of calculated NVE for $E^{201}Hg$ versus $E^{199}Hg$, $E^{200}Hg$ and $E^{204}Hg$ for individual Hg species relative to Hg(0) vapor (Table S9.5). Data is based on nuclear charge radii from Fricke & Heilig[53], Angeli-2013[54], and Nadjakov[55] and computational data by Wiederhold et al. [40] for the contributions of the NVE to MIF.

S10 Calculation of enrichment factors (ε, E)

The magnitude of isotope fractionation between the substrate (S) and the product (P) can be described by the isotopic fractionation factor α:[56]

$$\alpha_{S-P} = \frac{R_S}{R_P} \quad (S10.1)$$

where R_A and R_B are the isotope ratios in the substrate and the product phase, respectively. Similar to the δ notation, α values are frequently transformed to per mill (‰) values and reported as isotopic enrichment factors ε:[56]

$$\varepsilon_{S-P} = (\alpha_{S-P} - 1) * 1000 \quad (S10.2)$$

Rayleigh distillation occurs in the case of a reaction (kinetic or equilibrium) where the substrate does not continue to exchange with the product, producing a more pronounced isotope fractionation in the remaining substrate. In these cases, the enrichment factor is constant between the substrate and the instantaneous product, but the contrast between substrate and cumulative product increases with further progress of the reaction.[56,57] For kinetic reduction experiments a Rayleigh model was fitted using equations S10.3 – S10.5[38,56]

$$\delta_s = \delta_0 + \varepsilon * \ln(f) \quad (S10.3)$$

$$\delta_{pi} = \delta_s + \varepsilon \quad (S10.4)$$

$$\delta_{pc} = \delta_s - \frac{\varepsilon * \ln(f)}{(1 - f)} \quad (S10.5)$$

where δ_0 is the initial δ^{202}Hg value of the added Hg, f is the fraction of Hg(II) remaining in solution, δ_s, δ_{pi} and δ_{pc} are the isotope values for the substrate, instantaneous product and cumulative product, respectively.

Equilibrium isotope fractionation happens during isotope exchange when the forward and backward reaction rates of the isotopes that lead to isotope redistribution are identical and the substrate and product undergo complete isotopic exchange during the process (closed-system equilibrium).[57] Equilibrium fractionation of Hg isotopes was modeled fitting the measured δ^{202}Hg of reactors and traps to linear functions for a closed system with accumulated product following equations S10.6 – S10.7[57]

$$\delta_s = \delta_0 + \varepsilon * (1 - f) \quad (S10.6)$$

$$\delta_{pc} = \delta_0 + \varepsilon * f \quad (S10.7)$$

Enrichment factors for mass-independent fractionation (E) were calculated in the same way as ε using the respective Δ-values instead of δ-values in equations S10.3 – S10.7.

S11 Comparison of enrichment factors derived from different model approaches

Table S11. Comparison of the enrichment factors (ε^{202}Hg, E^{199}Hg, E^{201}Hg) for model fits minimizing the sum of squared residuals (SSR) for reactors only (R), traps only (T) or both reactors and traps (R & T). Additionally, enrichment factors determined based on linearized Rayleigh plots (ln(f) vs. δ or ln(f) vs. Δ) are reported, where the slope of linear regressions of reactor samples and the intercept of linear regressions of trap samples indicate ε. For a comparison the determined ε for linear equilibrium models are added to open system experiments and a Rayleigh fit is added to the closed system experiment, similar to Figure 1 and Figure 2. The goodness of fit was assessed by comparing the SSR of fits for the Rayleigh (R&T) model using reactors and traps to the respective equilibrium (R&T) model fit and are reported as the ratio of SSRs where values >1 indicate a better fit (lower SSR) for equilibrium fits and values <1 better fits for Rayleigh models. Please note that these values can only be regarded as a semi-quantitative relative estimate of the goodness of fit because the number of data points and their relative fraction reacted were different between the experiments. Fits for ^{200}Hg and ^{204}Hg were not included into the model comparison due to the low extent of the even mass MIF.

**Experiment**		**Least sum of squares (Excel solver)**			**Linear regression (linearized Rayleigh plots)**		**Linear equilibrium fit**			**Ratio of $SSR_{Rayleigh}$ / $SSR_{equilibrium}$**
		R & T	R	T	slope R	intercept T	R & T	R	T	
Fe(II) kinetic 0.5 mM Cl-	ε^{202}Hg	-2.20	-2.09	-2.54	-2.09	-2.49	-3.85	-4.55	-2.73	0.59
	E^{199}Hg	0.21	0.19	0.27	0.19	0.25	0.34	0.42	0.30	0.45
	E^{201}Hg	0.13	0.12	0.17	0.12	0.15	0.21	0.27	0.17	0.31
Fe(II) kinetic 10 mM Cl-	ε^{202}Hg	-2.14	-2.13	-2.15	-2.13	-2.38	-3.33	-3.95	-2.67	0.80
	E^{199}Hg	0.24	0.23	0.25	0.28	0.28	0.38	0.47	0.30	0.75
	E^{201}Hg	0.15	0.15	0.15	0.17	0.17	0.23	0.30	0.18	0.22
Fe(II) isotope exchange	ε^{202}Hg	-2.13	-2.13	-2.15	-2.31	-2.26	-2.44	-2.95	-2.37	4.81
	E^{199}Hg	0.24	0.23	0.25	0.26	0.34	0.34	0.32	0.34	3.01
	E^{201}Hg	0.15	0.15	0.17	0.17	0.21	0.21	0.22	0.21	1.51
Fe(II)-boehmite	ε^{202}Hg	-2.10	-1.81	-2.27	-1.81	-2.36	-2.73	-2.48	-2.56	1.25
	E^{199}Hg	0.19	0.18	0.19	0.18	0.29	0.24	0.25	0.32	0.97
	E^{201}Hg	0.14	0.12	0.14	0.12	0.17	0.19	0.16	0.19	2.08
Fe(II)-goethite (pH 6.5)	ε^{202}Hg	-2.09	-1.94	-2.25	-1.94	-2.31	-2.92	-1.93	-2.98	1.01
	E^{199}Hg	0.24	0.22	0.27	0.22	0.27	0.34	0.21	0.36	0.89
	E^{201}Hg	0.16	0.15	0.17	0.15	0.17	0.22	0.18	0.17	0.96
Magnetite	ε^{202}Hg	-1.38	-1.34	-1.79	-1.34	-1.53	-3.09	-3.51	-1.84	0.19
	E^{199}Hg	0.13	0.13	0.13	0.13	0.17	0.30	0.33	0.20	0.48
	E^{201}Hg	0.09	0.08	0.08	0.08	0.09	0.19	0.22	0.12	0.17

S12 Statistical testing

Table S12. Results of a paired, two-sided Wilcoxon signed rank test used to test whether there was a significant difference between the measured values of traps and reactors for $\Delta^{199}Hg$, $\Delta^{200}Hg$, $\Delta^{201}Hg$, and $\Delta^{204}Hg$ of each experiment. The confidence level was set at 95%.

Experiment		Pseudo-median difference	95 % confidence interval		p-value	r coefficient
Fe(II) 0.5 mM Cl (n=10)	$\Delta^{199}Hg$	-0.3693	-0.4398	-0.2984	0.0020	0.88 (L)
	$\Delta^{200}Hg$	-0.0587	-0.0778	-0.0384	0.0020	0.88 (L)
	$\Delta^{201}Hg$	-0.2366	-0.2766	-0.1854	0.0020	0.88 (L)
	$\Delta^{204}Hg$	0.0462	0.0057	0.0852	0.0273	0.69 (L)
Fe(II) 10 mM Cl (n=19)	$\Delta^{199}Hg$	-0.3712	-0.4130	-0.3377	3.82E-06	0.88 (L)
	$\Delta^{200}Hg$	-0.0570	-0.0636	-0.0450	3.92E-06	0.88 (L)
	$\Delta^{201}Hg$	-0.2265	-0.2572	-0.2043	3.82E-06	0.88 (L)
	$\Delta^{204}Hg$	0.0461	0.0314	0.0601	2.67E-05	0.84 (L)
Fe(II) isotope exchange (n=14)	$\Delta^{199}Hg$	-0.3392	-0.3663	-0.3103	0.0001	0.88 (L)
	$\Delta^{200}Hg$	-0.0360	-0.0564	-0.0284	0.0001	0.88 (L)
	$\Delta^{201}Hg$	-0.2122	-0.2337	-0.1910	0.0001	0.88 (L)
	$\Delta^{204}Hg$	0.0501	0.0328	0.0677	0.0002	0.86 (L)
Magnetite (n=6)	$\Delta^{199}Hg$	-0.1800	-0.2301	-0.1100	0.0355	0.90 (L)
	$\Delta^{200}Hg$	-0.0200	-0.0401	0.0099	0.2807	0.48 (M)
	$\Delta^{201}Hg$	-0.1200	-0.1600	-0.0100	0.0313	0.90 (L)
	$\Delta^{204}Hg$	0.0100	-0.1200	0.1000	0.8438	0.13 (S)
Fe(II)-boehmite (n=9)	$\Delta^{199}Hg$	-0.2927	-0.3069	-0.2628	0.0039	0.89 (L)
	$\Delta^{200}Hg$	-0.0445	-0.0573	-0.0348	0.0039	0.89 (L)
	$\Delta^{201}Hg$	-0.1754	-0.1971	-0.1666	0.0039	0.89 (L)
	$\Delta^{204}Hg$	0.0070	-0.0235	0.0290	0.5703	0.22 (S)
Fe(II)-goethite (pH 6.5) (n=12)	$\Delta^{199}Hg$	-0.2500	-0.3303	-0.1567	0.0005	0.88 (L)
	$\Delta^{200}Hg$	-0.0310	-0.0470	-0.0115	0.0093	0.72 (L)
	$\Delta^{201}Hg$	-0.1651	-0.2122	-0.1170	0.0005	0.88 (L)
	$\Delta^{204}Hg$	0.0134	-0.0049	0.0328	0.1514	0.43 (M)

n: Number of pairwise differences.

The test was performed in R using the package *stats* version 4.2.2.[58] The effect size (r coefficient) was calculated according to Cohen (1992).[59]

	Coefficient r
Small effect (S)	< 0.3
Medium effect (M)	0.3 to 0.5
Large effect (L)	> 0.5

References

(1) Bloom, N. S.; Preus, E.; Katon, J.; Hiltner, M. Selective Extractions to Assess the Biogeochemically Relevant Fractionation of Inorganic Mercury in Sediments and Soils. *Anal. Chim. Acta* **2003**, *479* (2), 233–248. DOI 10.1016/S0003-2670(02)01550-7.

(2) EPA. *Method 1631, Revision E: Mercury in Water by Oxidation, Purge and Trap, and Cold Vapor Atomic Fluorescence Spectrometry*; United States Environmental Protection Agency, 2002. https://www.nemi.gov/methods/method_summary/9628/ (accessed 2021-09-02).

(3) Ferreira, C. M. H.; Pinto, I. S. S.; Soares, E. V.; Soares, H. M. V. M. (Un)Suitability of the Use of pH Buffers in Biological, Biochemical and Environmental Studies and Their Interaction with Metal Ions-a Review. *RSC Adv.* **2015**, *5* (39), 30989–31003. DOI 10.1039/c4ra15453c.

(4) Kandegedara, A.; Rorabacher, D. B. Noncomplexing Tertiary Amines as "better" Buffers Covering the Range of pH 3-11. Temperature Dependence of Their Acid Dissociation Constants. *Anal. Chem.* **1999**, *71* (15), 3140–3144. DOI 10.1021/ac9902594.

(5) Yu, Q.; Kandegedara, A.; Xu, Y.; Rorabacher, D. B. Avoiding Interferences from Good's Buffers: A Contiguous Series of Noncomplexing Tertiary Amine Buffers Covering the Entire Range of pH 3-11. *Anal. Biochem.* **1997**, *253* (1), 50–56. DOI 10.1006/abio.1997.2349.

(6) Charlet, L.; Bosbach, D.; Peretyashko, T. *Natural Attenuation of TCE, As, Hg Linked to the Heterogeneous Oxidation of Fe(II): Chem. Geol.*; 2002; 190(1), 303–319. DOI 10.1016/S0009-2541(02)00122-5.

(7) Amirbahman, A.; Kent, D. B.; Curtis, G. P.; Marvin-Dipasquale, M. C. Kinetics of Homogeneous and Surface-Catalyzed Mercury(II) Reduction by Iron(II). *Environ. Sci. Technol.* **2013**, *47* (13), 7204–7213. DOI 10.1021/es401459p.

(8) O'Loughlin, E. J.; Boyanov, M. I.; Kemner, K. M.; Thalhammer, K. O. Reduction of Hg(II) by Fe(II)-Bearing Smectite Clay Minerals. *Minerals* **2020**, *10* (12), 1–13. DOI 10.3390/min10121079.

(9) Bone, S. E.; Bargar, J. R.; Sposito, G. Mackinawite (FeS) Reduces Mercury(II) under Sulfidic Conditions. *Environ. Sci. Technol.* **2014**, *48* (18), 10681–10689. DOI 10.1021/es501514r.

(10) Dideriksen, K.; Frandsen, C.; Bovet, N.; Wallace, A. F.; Sel, O.; Arbour, T.; Navrotsky, A.; De Yoreo, J. J.; Banfield, J. F. Formation and Transformation of a Short Range Ordered Iron Carbonate Precursor. *Geochim. Cosmochim. Acta* **2015**, *164*, 94–109. DOI 10.1016/j.gca.2015.05.005.

(11) Viollier, E.; Inglett, P. W.; Hunter, K.; Roychoudhury, A. N.; Van Cappellen, P. The Ferrozine Method Revisited: Fe(II)/Fe(III) Determination in Natural Waters. *Appl. Geochem.* **2000**, *15* (6), 785–790. DOI 10.1016/S0883-2927(99)00097-9.

(12) Stookey, L. L. Ferrozine - A New Spectrophotometric Reagent for Iron. *Anal. Chem.* **1970**, *42* (7), 779–781. DOI 10.1021/ac60289a016.

(13) Nordin, J.; Persson, P.; Laiti, E.; Sjöberg, S. Adsorption of O-Phthalate at the Water-Boehmite (γ-AlOOH) Interface: Evidence for Two Coordination Modes. *Langmuir* 1997, 13(15), 4085–4093. . DOI 10.1021/la970066g.

(14) Nordin, J.; Persson, P.; Nordin, A.; Sjöberg, S. Inner-Sphere and Outer-Sphere Complexation of a Polycarboxylic Acid at the Water-Boehmite (γ-AlOOH) Interface: A Combined Potentiometric and IR Spectroscopic Study. *Langmuir* **1998**, *14* (13), 3655–3662. DOI 10.1021/la9712449.

(15) Laiti, E.; Persson, P.; Öhman, L. O. Balance between Surface Complexation and Surface Phase Transformation at the Alumina/Water Interface. *Langmuir* **1998**, *14* (4), 825–831. DOI 10.1021/la970383n.
(16) Schwertmann, U.; Cornell, R. M. *Iron Oxides in the Laboratary*; WILEY-VCH Verlag GmbH, Weinheim 2000; DOI 10.1002/9783527613229.
(17) Coppi, M. V.; Leang, C.; Sandler, S. J.; Lovley, D. R. Development of a Genetic System for Geobacter Sulfurreducens. *Appl. Environ. Microbiol.* **2001**, *67* (7), 3180–3187. DOI 10.1128/AEM.67.7.3180-3187.2001.
(18) Schwertmann, U.; Cornell, R. M. *Iron Oxides in the Laboratary*; WILEY-VCH Verlag GmbH, Weinheim 2000;. DOI 10.1002/9783527613229.
(19) Byrne, J. M.; Muhamadali, H.; Coker, V. S.; Cooper, J.; Lloyd, J. R. Scale-up of the Production of Highly Reactive Biogenic Magnetite Nanoparticles Using Geobacter Sulfurreducens. *J. R. Soc. Interface* **2015**, *12* (107). DOI 10.1098/rsif.2015.0240.
(20) Cutting, R. S.; Coker, V. S.; Telling, N. D.; Kimber, R. L.; Pearce, C. I.; Ellis, B. L.; Lawson, R. S.; Van Gerrit Laan, D. E. R.; Pattrick, R. A. D.; Vaughan, D. J.; Arenholz, E.; Lloyd, J. R. Optimizing Cr(VI) and Tc(VII) Remediation through Nanoscale Biomineral Engineering. *Environ. Sci. Technol.* **2010**, *44* (7), 2577–2584. DOI 10.1021/es902119u.
(21) Gustafsson, J. P. Visual MINTEQ 3.1 User Guide. *Department of Land and Water Resources, Stockholm, Sweden*, 2011, 1–73.
(22) Stumm, W.; Morgan, J. J. *Aquatic Chemistry - Chemical Equilibria and Rates in Natural Waters*; John Wiley & Sons, 1996.
(23) Allard, B.; Arsenie, I. Abiotic Reduction of Mercury by Humic Substances in Aquatic System - an Important Process for the Mercury Cycle. *Water. Air. Soil Pollut.* **1991**, *56* (1), 457–464. DOI 10.1007/BF00342291.
(24) Blum, J. D.; Bergquist, B. A. Reporting of Variations in the Natural Isotopic Composition of Mercury. *Anal. Bioanal. Chem.* **2007**, *388* (2), 353–359. DOI 10.1007/s00216-007-1236-9.
(25) Brocza, F. M.; Biester, H.; Richard, J.-H.; Kraemer, S. M.; Wiederhold, J. G. Mercury Isotope Fractionation in the Subsurface of a Hg(II) Chloride-Contaminated Industrial Legacy Site. *Environ. Sci. Technol.* **2019**, *53* (13), 7296–7305. DOI 10.1021/acs.est.9b00619.
(26) Goix, S.; Maurice, L.; Laffont, L.; Rinaldo, R.; Lagane, C.; Chmeleff, J.; Menges, J.; Heimbürger, L. E.; Maury-Brachet, R.; Sonke, J. E. Quantifying the Impacts of Artisanal Gold Mining on a Tropical River System Using Mercury Isotopes. *Chemosphere* **2019**, *219*, 684–694. DOI 10.1016/j.chemosphere.2018.12.036.
(27) Grigg, A. R. C.; Kretzschmar, R.; Gilli, R. S.; Wiederhold, J. G. Mercury Isotope Signatures of Digests and Sequential Extracts from Industrially Contaminated Soils and Sediments. *Sci. Total Environ.* **2018**, *636*, 1344–1354. DOI 10.1016/j.scitotenv.2018.04.261.

(28) Jiskra, M.; Marusczak, N.; Leung, K. H.; Hawkins, L.; Prestbo, E.; Sonke, J. E. Automated Stable Isotope Sampling of Gaseous Elemental Mercury (ISO-GEM): Insights into GEM Emissions from Building Surfaces. *Environ. Sci. Technol.* **2019**, *53* (8), 4346–4354. DOI 10.1021/acs.est.8b06381.
(29) Jiskra, M.; Wiederhold, J. G.; Skyllberg, U.; Kronberg, R.-M. M.; Kretzschmar, R. Source Tracing of Natural Organic Matter Bound Mercury in Boreal Forest Runoff with Mercury Stable Isotopes. *Environ. Sci. Process. Impacts* **2017**, *19* (10), 1235–1248. DOI 10.1039/c7em00245a.
(30) Obrist, D.; Agnan, Y.; Jiskra, M.; Olson, C. L.; Colegrove, D. P.; Hueber, J.; Moore, C. W.; Sonke, J. E.; Helmig, D. Tundra Uptake of Atmospheric Elemental Mercury Drives Arctic Mercury Pollution. *Nature* **2017**, *547* (7662), 201–204. DOI 10.1038/nature22997.
(31) Smith, R. S.; Wiederhold, J. G.; Jew, A. D.; Brown, G. E.; Bourdon, B.; Kretzschmar, R. Stable Hg Isotope Signatures in Creek Sediments Impacted by a Former Hg Mine. *Environ. Sci. Technol.* **2015**, *49* (2), 767–776. DOI 10.1021/es503442p.
(32) McLagan, D. S.; Schwab, L.; Wiederhold, J. G.; Chen, L.; Pietrucha, J.; Kraemer, S. M.; Biester, H. Demystifying Mercury Geochemistry in Contaminated Soil–Groundwater Systems with Complementary Mercury Stable Isotope, Concentration, and Speciation Analyses. *Environ. Sci. Process. Impacts* **2022**, *24*, 1406–1429. DOI 10.1039/d1em00368b.
(33) Brezonik, P. L.; Arnold, W. A. *Water Chemistry - An Introduction to the Chemistry of Natural and Engineered Aquatic Systems*; Oxford University Press: New York, 2011.
(34) Wiatrowski, H. A.; Das, S.; Kukkadapu, R.; Ilton, E. S.; Barkay, T.; Yee, N. Reduction of Hg(II) to Hg(0) by Magnetite. *Environ. Sci. Technol.* **2009**, *43* (14), 5307–5313. DOI 10.1021/es9003608.
(35) Etique, M.; Bouchet, S.; Byrne, J. M.; Arrigo, L. K. T.; Kaegi, R.; Kretzschmar, R. Mercury Reduction by Nanoparticulate Vivianite. *Environ. Sci. Technol.* **2021**, *55* (5), 3399–3407. DOI 10.1021/acs.est.0c05203.
(36) Ha, J.; Zhao, X.; Yu, R.; Barkay, T.; Yee, N. Hg(II) Reduction by Siderite (FeCO3). *Appl. Geochem.* **2017**, *78*, 211–218. DOI 10.1016/j.apgeochem.2016.12.017.
(37) Remy, P. P.; Etique, M.; Hazotte, A. A.; Sergent, A. S.; Estrade, N.; Cloquet, C.; Hanna, K.; Jorand, F. P. A. Pseudo-First-Order Reaction of Chemically and Biologically Formed Green Rusts with Hg^{II} and $C_{15}H_{15}N_3O_2$: Effects of pH and Stabilizing Agents (Phosphate, Silicate, Polyacrylic Acid, and Bacterial Cells). *Water Res.* **2015**, *70*, 266–278. DOI 10.1016/j.watres.2014.12.007.
(38) Wiederhold, J. G. Metal Stable Isotope Signatures as Tracers in Environmental Geochemistry. *Environ. Sci. Technol.* **2015**, *49* (5), 2606–2624. DOI 10.1021/es504683e.
(39) Schauble, E. A. Role of Nuclear Volume in Driving Equilibrium Stable Isotope Fractionation of Mercury, Thallium, and Other Very Heavy Elements. *Geochim. Cosmochim. Acta* **2007**, *71* (9), 2170–2189. DOI 10.1016/j.gca.2007.02.004.
(40) Wiederhold, J. G.; Cramer, C. J.; Daniel, K.; Infante, I.; Bourdon, B.; Kretzschmar, R. Equilibrium Mercury Isotope Fractionation between Dissolved Hg(II) Species and Thiol-Bound Hg. *Environ. Sci. Technol.* **2010**, *44* (11), 4191–4197. DOI 10.1021/es100205t.
(41) Bergquist, B. A.; Blum, J. D. The Odds and Evens of Mercury Isotopes: Applications of Mass-Dependent and Mass-Independent Isotope Fractionation. *Elements* **2009**, *5* (6), 353–357. DOI 10.2113/gselements.5.6.353.

(42) Yang, S.; Liu, Y. Nuclear Volume Effects in Equilibrium Stable Isotope Fractionations of Mercury, Thallium and Lead. *Sci. Rep.* **2015**, *5* (July), 1–12. DOI 10.1038/srep12626.
(43) Buchachenko, A. L. Magnetic Isotope Effect: Nuclear Spin Control of Chemical Reactions. *J. Phys. Chem. A* **2001**, *105* (44). DOI 10.1021/jp011261d.
(44) Motta, L. C.; Chien, A. D.; Rask, A. E.; Zimmerman, P. M. Mercury Magnetic Isotope Effect: A Plausible Photochemical Mechanism. *J. Phys. Chem. A* **2020**, *124* (19), 3711–3719. DOI 10.1021/acs.jpca.0c00661.
(45) Zheng, W.; Hintelmann, H. Isotope Fractionation of Mercury during Its Photochemical Reduction by Low-Molecular-Weight Organic Compounds. *J. Phys. Chem. A* **2010**, *114* (12), 4246–4253. DOI 10.1021/jp9111348.
(46) Chen, J. B.; Hintelmann, H.; Feng, X. B.; Dimock, B. Unusual Fractionation of Both Odd and Even Mercury Isotopes in Precipitation from Peterborough, ON, Canada. *Geochim. Cosmochim. Acta* **2012**, *90*, 33–46. DOI 10.1016/j.gca.2012.05.005.
(47) Gratz, L. E.; Keeler, G. J.; Blum, J. D.; Sherman, L. S. Isotopic Composition and Fractionation of Mercury in Great Lakes Precipitation and Ambient Air. *Environ. Sci. Technol.* **2010**, *44* (20), 7764–7770. DOI 10.1021/es100383w.
(48) Demers, J. D.; Blum, J. D.; Zak, D. R. Mercury Isotopes in a Forested Ecosystem: Implications for Air-Surface Exchange Dynamics and the Global Mercury Cycle. *Glob. Biogeochem. Cycles* **2013**, *27* (1), 222–238. DOI 10.1002/gbc.20021.
(49) Yuan, S.; Chen, J.; Hintelmann, H.; Cai, H.; Yuan, W.; He, S.; Zhang, K.; Zhang, Y.; Liu, Y. Event-Based Atmospheric Precipitation Uncovers Significant Even and Odd Hg Isotope Anomalies Associated with the Circumpolar Vortex. *Environ. Sci. Technol.* **2022**, *56* (17), 12713–12722. DOI 10.1021/acs.est.2c02613.
(50) Fu, X.; Jiskra, M.; Yang, X.; Marusczak, N.; Enrico, M.; Chmeleff, J.; Heimbürger-Boavida, L. E.; Gheusi, F.; Sonke, J. E. Mass-Independent Fractionation of Even and Odd Mercury Isotopes during Atmospheric Mercury Redox Reactions. *Environ. Sci. Technol.* **2021**, *55* (14), 10164–10174. DOI 10.1021/acs.est.1c02568.
(51) Sun, G.; Feng, X.; Yin, R.; Wang, F.; Lin, C. J.; Li, K.; Sommar, J. O. Dissociation of Mercuric Oxides Drives Anomalous Isotope Fractionation during Net Photo-Oxidation of Mercury Vapor in Air. *Environ. Sci. Technol.* **2022**, *56* (18), 13428–13438. DOI 10.1021/acs.est.2c02722.
(52) Vermeesch, P. IsoplotR: A Free and Open Toolbox for Geochronology. *Geosci. Front.* **2018**, *9* (5), 1479–1493. DOI 10.1016/j.gsf.2018.04.001.
(53) Fricke, G.; Heilig, K. Nuclear Charge Radii. In *Landolt-Börnstein - Numerical Data and Functional Relationships in Science and Technology*; Schopper, H., Ed.; Springer, 2004. DOI 10.1007/b87879.
(54) Angeli, I.; Marinova, K. P. Table of Experimental Nuclear Ground State Charge Radii: An Update. *At. Data Nucl. Data Tables* **2013**, *99* (1), 69–95. DOI 10.1016/j.adt.2011.12.006.
(55) Nadjakov, E. G.; Marinova, K. P.; Gangrsky, Y. P. Systematics of Nuclear Charge Radii. *At. Data Nucl. Data Tables* **1994**, *56* (1), 133–157. DOI 10.1016/0375-9474(79)90604-3.
(56) Hunkeler, D.; Elsner, M. Principles and Mechanisms of Isotope Fractionation. In *Environmental Isotopes in Biodegradation and Bioremediation*; Aelion, C.M., Höhener, P., Hunkeler, D., Aravena, R., Eds.; CRC Press: Boca Raton 2009; pp 57–92. DOI 10.1201/9781420012613-9.

(57) Johnson, C. M.; Beard, B. L.; Albarède, F. Overview and General Concepts. *Rev. Mineral. Geochem.* **2004**, *55*, 1–24. DOI 10.2138/gsrmg.55.1.1.
(58) R Core Team. R: A Language and Environment for Statistical Computing, 2021. https://www.r-project.org/.
(59) Cohen, J. Statistical Power Analysis. *Curr. Dir. Psychol. Sci.* **1992**, *1* (3), 98–101. DOI 10.1111/1467-8721.ep10768783.
